# What is “hospital resilience”? A scoping review on conceptualization, operationalization, and evaluation

**DOI:** 10.3389/fpubh.2022.1009400

**Published:** 2022-10-14

**Authors:** Merette Khalil, Hamid Ravaghi, Dalia Samhouri, John Abo, Ahmed Ali, Hala Sakr, Alex Camacho

**Affiliations:** ^1^Department for Universal Health Coverage and Health Systems, World Health Organization Regional Office for the Eastern Mediterranean, Cairo, Egypt; ^2^Health Emergencies Programme, World Health Organization, Regional Office for the Eastern Mediterranean, Cairo, Egypt; ^3^Asian Disaster Preparedness Center, Bangkok, Thailand; ^4^Department of Healthier Populations, World Health Organization, Regional Office for the Eastern Mediterranean, Cairo, Egypt; ^5^Health Emergencies Programme, World Health Organization, Regional Office for the Americas, Washington, DC, United States

**Keywords:** hospitals, resilience, health emergencies, health systems, adaptive leadership

## Abstract

**Background:**

COVID-19 underscored the importance of building *resilient* health systems and hospitals. Nevertheless, evidence on hospital resilience is limited without consensus on the concept, its application, or measurement, with practical guidance needed for action at the facility-level.

**Aim:**

This study establishes a baseline for understanding hospital resilience, exploring its 1) conceptualization, 2) operationalization, and 3) evaluation in the empirical literature.

**Methods:**

Following Arksey and O'Malley's model, a scoping review was conducted, and a total of 38 articles were included for final extraction.

**Findings and discussion:**

In this review, hospital resilience is conceptualized by its components, capacities, and outcomes. The interdependence of six components (1) space, 2) stuff, 3) staff, 4) systems, 5) strategies, and 6) services) influences hospital resilience. Resilient hospitals must absorb, adapt, transform, and learn, utilizing all these capacities, sometimes simultaneously, through prevention, preparedness, response, and recovery, within a risk-informed and all-hazard approach. These capacities are not static but rather are dynamic and should improve continuously occur over time. Strengthening hospital resilience requires both hard and soft resilience. Hard resilience encompasses the structural (or constructive) and non-structural (infrastructural) aspects, along with agility to rearrange the space while hospital's soft resilience requires resilient staff, finance, logistics, and supply chains (stuff), strategies and systems (leadership and coordination, community engagement, along with communication, information, and learning systems). This ultimately results in hospitals maintaining their function and providing quality and continuous critical, life-saving, and essential services, amidst crises, while leaving no one behind. Strengthening hospital resilience is interlinked with improving health systems and community resilience, and ultimately contributes to advancing universal health coverage, health equity, and global health security. The nuances and divergences in conceptualization impact how hospital resilience is applied and measured. Operationalization and evaluation strategies and frameworks must factor hospitals' evolving capacities and varying risks during both routine and emergency times, especially in resource-restrained and emergency-prone settings.

**Conclusion:**

Strengthening hospital resilience requires consensus regarding its conceptualization to inform a roadmap for operationalization and evaluation and guide meaningful and effective action at facility and country level. Further qualitative and quantitative research is needed for the operationalization and evaluation of hospital resilience comprehensively and pragmatically, especially in fragile and resource-restrained contexts.

## Background

The COVID-19 pandemic exposed global gaps in emergency preparedness, highlighted the power of technological innovations and agility in times of crises, and reinforced the importance of resilience spanning individual, community, organizational, and systemic levels (1, 2). Building *resilient* health systems and hospitals is critical to advance universal health coverage (UHC) and global health security (3, 4). Health systems resilience can be defined as the ability to “resist, absorb, accommodate, adapt to, transform and recover in a timely and efficient manner”; while often conceptualized within the context of health emergencies and disaster risk management (HEDRM), resilience may also be to other social, political, economic and environmental shocks (5, 6). In the last decade, scholars presented numerous frameworks to understand and strengthen health systems resilience to various types of external shocks, ranging from climate-related disasters to infectious disease outbreaks to political unrest and financial crashes (5–7). Health systems resilience has been defined as a process (6, 8), a capacity (5), an ability (9), an outcome (6, 10), attributes (11, 12), and as a policy objective (4). Despite the growing literature on this subject, this nascent topic is still under-researched, with varying and complex definitions limiting its operationalization (6, 8, 13). These complexities and divergences in definitions make it difficult to translate the concept into practice at the system-level with even greater challenges at the hospital-level.

Hospitals are miniature health systems, complex organizations utilizing innovative technologies and infrastructures, comprised of and serving diverse communities (14). Hospitals have been the backbone of the COVID-19 response, adapting to continue providing critical and essential services, fight the pandemic and manage complex emergencies, especially in fragile, conflict and resource-restrained settings (FCRS) (14, 15). In these settings, hospitals face exacerbated health systems pressures, such as political unrest; violence; fragmentation in governance, service delivery, policy and decision-making; disruptions and shortages of critical human, material, medical, and financial resources and supply chains; infrastructural damages; impeding the delivery of safe, continuous, high-quality, patient-centered services (14, 16). Within these contexts, it can be argued that hospitals express an “everyday resilience” to chronic health systems shocks and emergencies, with many lessons learned from hospitals responses to COVID-19, especially in FCRS (3). The pandemic confirmed that hospitals face and respond to numerous hazards simultaneously while adapting to various technological and social changes, highlighting the need for practical and systematic guidance on strengthening hospital resilience, during both routine and emergency times.

Hospital resilience has been explored across various disciplines, from HEDRM to engineering, in efforts to strengthen hospital safety and functionality (10, 17, 18). Extrapolations from these various fields can be used to define hospital resilience, including but not limited to conceptualizations from health systems, organizational, engineering, physiological, disaster, and community resilience (5, 8). Nevertheless, adaptation and synthesis are required to translate these complex and multi-dimensional concepts into practice at the facility-level. Hospital resilience is a novel concept in global health with limited and divergent definitions; there is no broad consensus on a comprehensive conceptual framework further limiting its application and evaluation (19). To inform actionable guidance for hospital managers on operationalizing and evaluating hospital resilience, it is necessary to disentangle these complexities in definitions and reach a common understanding and consensus around the concept of “hospital resilience.”

This review provides a starting point for understanding how hospital resilience is 1) conceptualized, 2) operationalized, and 3) evaluated in the empirical literature. On its basis, the paper informs the development of a practical and pilotable guidance for hospital managers as part of a larger regional project on *Strengthening Hospital Resilience in the Eastern Mediterranean Region (EMR)*.

## Methods

**Figure 1:**
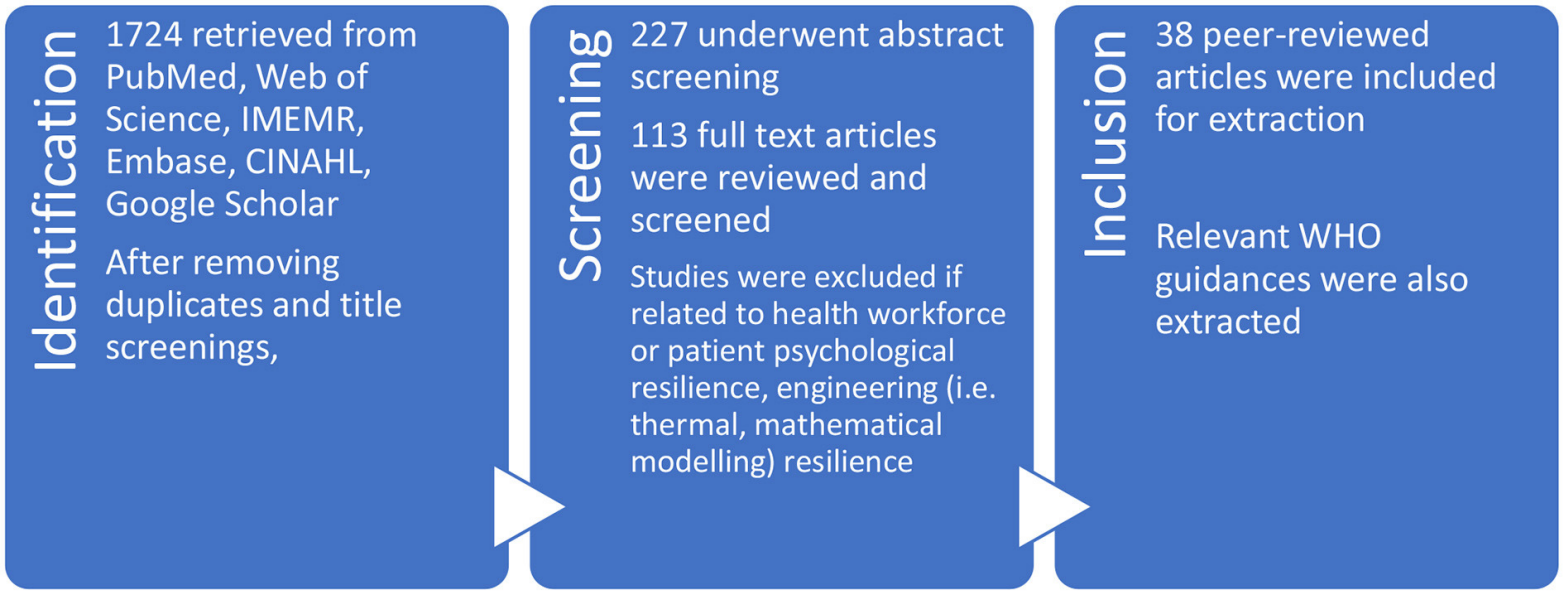
PRISMA flow diagram.

Due to the novelty of the subject, a scoping review was appropriately utilized to achieve the following four objectives: 1) identify the types of existing evidence related to “**hospital resilience**,” 2) clarify and compare the key concepts, definitions, and components, 3) compile experiences, pre-requisites, and tips for its operationalization, and 4) identify tools and/or strategies for its evaluation. Guided by Arksey and O'Malley's framework for scoping reviews (20), the following steps were completed:

1. **Identify the research question:** How is “hospital resilience” conceptualized, applied, and measured?
2. **Identify relevant studies:** The search strategy (Annex 1) was developed and revised through studying relevant systematic and scoping reviews on hospital and health systems resilience to identify and cross-check keywords, MeSH terms, and databases searched. Both peer-reviewed and gray literature were searched, using the following keywords and their variations: “hospitals” AND “resilience.” A review of reference lists of included studies and snowballing were conducted to identify other relevant literature. Relevant WHO guidance documents were also reviewed and extracted for this study.
3. **Study selection**: Systematic reviews on both health systems and hospital resilience indicated an introduction of the concept around 2011, as such searches were limited to the last 10 years to remain relevant with recent evidence. Zotero reference manager was used to remove duplicates and organize the articles. Articles screening occurred at three stages: title, abstract, and full-text, eliminating non-relevant articles (Figure 1). While limited by time and financial constraints, articles were screened in 3 UN languages, Arabic, English and French, most non-English entries were duplicates to their English counterparts. Articles that were not specifically related to “hospital resilience” were excluded, including articles related to: health workforce or patient's psychological resilience, organizational or community resilience which do not specifically mention hospitals, and environmental, thermal, infrastructural, or urban resilience which only addressed hospital's engineering, construction, and design aspects.

Systematic reviews on hospital and health systems resilience revealed that most studies were concentrated in the Global North. To counteract this limitation and shift the emphasis and contextualization toward developing and resource-restrained settings, all articles from low- and middle-income countries (LMIC) related to hospital resilience were included in the initial screening stages.

Articles related to health systems resilience were initially excluded but due to the dearth of literature, the reference lists of systematic and scoping reviews on health systems resilience were reviewed and relevant articles on hospitals were extracted and included. Systematic reviews on hospital preparedness were also screened as historically, the concept of hospital resilience was often described using this terminology.

4. **Chart the data**: Included entries was extracted on Microsoft Excel answering the four main scoping review objectives. The extraction table (Annex 2) included: Authors, Year, Country of origin, journal, Aim/purpose, Methodology, Setting, and the main findings, challenges, and tools, indicators, or frameworks for each of the three main sections: 1)conceptualization/definitions/components, 2) application/implementation/operationalization, and 3) evaluation/assessment/measurement.
5. **Summarize/report**: The results are organized according to four sections: a descriptive analysis of findings, followed by Hospital Resilience Conceptualization, Operationalization, and Evaluation.
6. **Expert consultation** was convened by the WHO/EMRO team presenting the findings of this review among multi-disciplinary and multi-specialty stakeholders to discuss, validate, and reach consensus on hospital resilience conceptualization, and interventions for operationalization and evaluation. As part of the larger mixed-methods research, the findings of this review were triangulated with qualitative data from key informant interviews and an online survey.

## Findings

### Descripive analysis of results

Regarding peer-reviewed publications, an initial 1,724 articles were identified; after removing duplicates and title screenings, 227 remained. Following abstract screenings, 113 articles underwent full-text screenings and a total of 38 entries specifically on hospital resilience were included for extraction. This is consistent with the included article ranges in other systematic reviews on hospital resilience (18, 21, 22).

**Figure 2:**
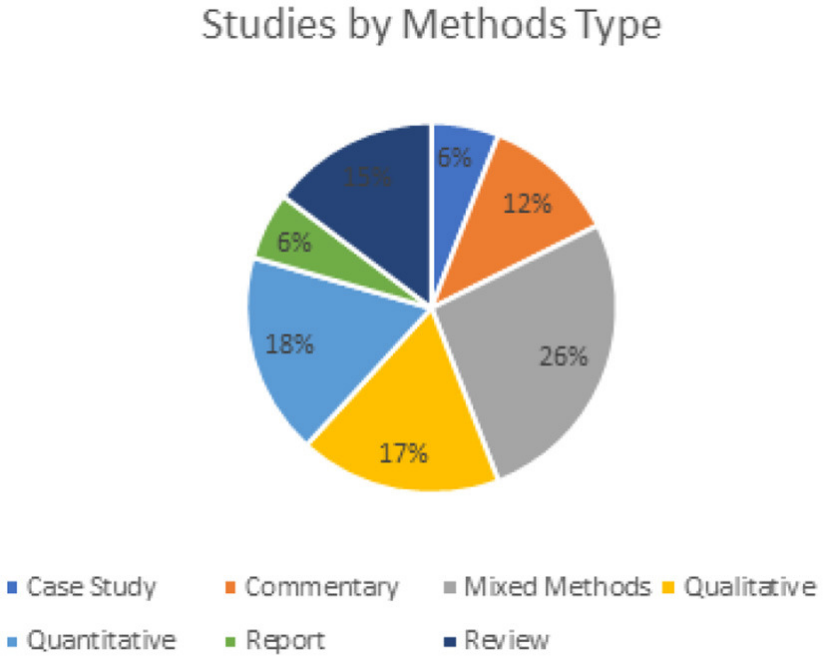
Studies by methods type.

A quarter (10/38) of the studies included in this review utilized mixed methods, and approximately equal studies utilized either qualitative and quantitative methods (Figure 2).

This scoping review found that 75% (29/38) of included articles were published after 2017, further confirming the newness of this topic in the empirical literature. This is further confirmed by a systematic review on “hospitals resilience” published in 2021, which found that 63% of articles were published between 2016-2019, with over a quarter published in 2019 (18). This is consistent with evidence on health systems resilience where a systematic review found that 62% of articles were published after 2017 (6).

**Figure 3:**
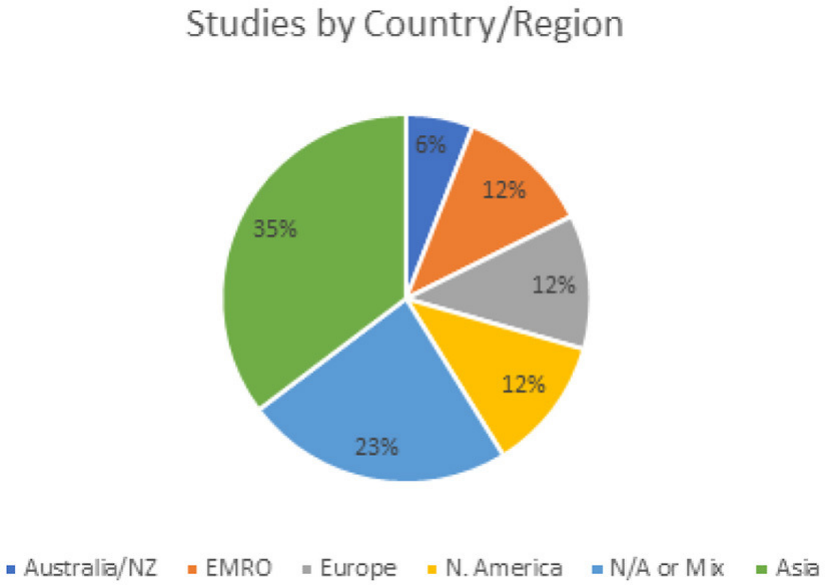
Geographical distribution of studies.

When comparing three systematic reviews on hospital resilience, they consistently reveal that the majority of articles published are from North America, indicating a limitation in contextualized evidence to FCRS such as the EMR where most countries are LMICs and more than half of countries facing humanitarian emergencies (18, 21, 22). This is in line with a systematic review on health systems resilience which found that only 25 and 18% were from LMIC respectively (6). A quarter of studies (9/38) in this review mentioned mixed or multiple countries, mostly systematic and scoping reviews, about a third (11/38) were from highly developed countries and regions including Europe, North America, Australia, and New Zealand, while a third (13/38) were from countries in Asia, most commonly Malaysia (Figure 3). Only 12% of studies (5/38) were from the EMR all of which were from Iran. The limited evidence from South America and African regions presents a geographical limitation and bias.

**Figure 4:**
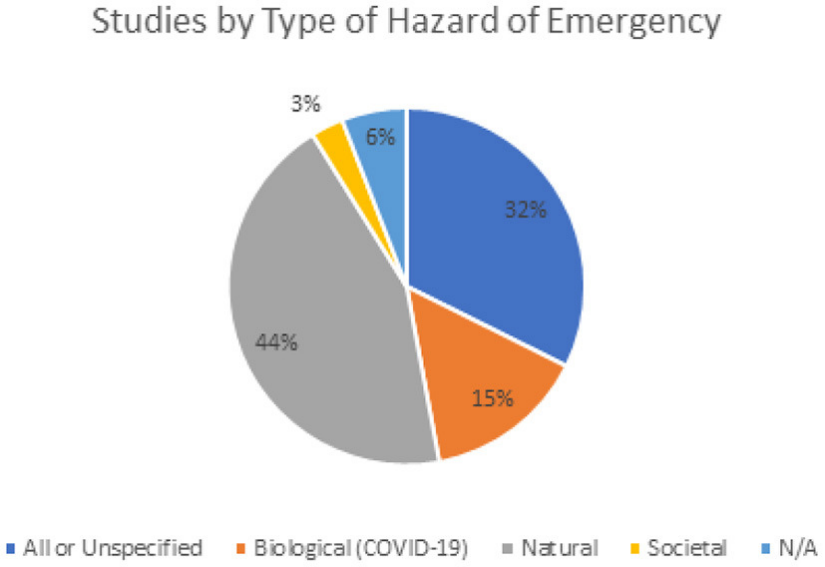
Studies by type of hazard or emergency.

Regarding the types of disasters, this study found that one-third of the articles did not specify the type of hazard or mention all types of disasters and hazards, 44% of including articles mentioned natural hazards, while only 15% mentioned biological hazards (Figure 4). All articles mentioning biological hazards were related to COVID-19. In Fallah-Aliabadi et al.'s systematic review, half of included articles utilized an all-hazard approach, on the other hand, another systematic review found that about 70% of included articles discussed specific disasters related to their hospital resilience (18, 21). This is similar to findings by Biddle et al., where 82% of included articles addressed a specific crisis or challenge at the health systems' level (6).

To complement the findings of these 38 articles specifically on hospital resilience, an additional 15 documents were also reviewed using the same tool (see methods, step 4); these included systematic reviews on health systems resilience and hospital preparedness along with gray literature, namely WHO or UN guidance documents and relevant publications on health systems and/or hospital resilience.

### Conceptualization

The literature displays wide divergences in conceptualizations of hospital resilience, and scholars and experts do not agree on a specific definition. The most significant challenge comes from defining the “elements,” “components” or “dimensions” of resilient hospitals. In defining hospital resilience, scholars across the literature have utilized a variety of terminologies for each of these categories. The diversity in language and terminologies, inconsistent categorizations, and varied conceptualizations of hospital resilience made it difficult to definitively name and organize this section and its sub-headings (Annexes 2 and 3).

In attempts to organize this section, reflect the richness and diversity of the literature, and capture the various elements of these definitions, the findings were charted according to three categories: 1) Use X, 2) To-do X, 3) Resulting-in X (Table 1). In this synthesis and for ease of organization, the authors have used the term “*components”* for the first category, “*capacities”* for the second, and “*outcomes”* for the third. Generally, the literature on hospital resilience is consistent in the third category (outcomes). Common themes are found regarding capacities. However, the most variation and diversity, including terminologies and concepts, are found for components. Therefore, the subtitles below are organized in this order from the most agreeable category, outcomes, to the most diverse one, components.

**Table 1:** Diversity in hospital resilience conceptualizations and terminologies.

**Components**	**Capacities**	**Outcomes & impacts**	**“Resilience as”**
**Using “X”**	**To do “X”**	**Resulting in “X”**	
“inputs”, “domains”, “factors”, “areas”, “domains”, “categories” “components”, “elements” “dimensions”, “building blocks”, “tools”, “pre-requisites”	“actions”, “activities”, “capacities”, “processes”	“outcomes”, “attributes”, “intermediate objectives”, “outputs”, and “impacts”	“robustness”, “toughness”, “elasticity”, “adaptability”, “agility”, “anti-fragility”, “business continuity”, “services continuity”, “emergency preparedness”, “crisis management”, “perseverance”

#### Outcomes

According to the literature, the most immediate and primary outcome of resilient hospitals is **maintain their functions and ensure a continuity of high-quality essential and critical services to vulnerable populations** (18); additional functions also include contributions to educational/research, social and economic aspects of communities (11, 14) (Annex 3). The WHO's framework on climate-resilient hospitals highlights five hospital resilience outcomes: Collapse, Recover worse than before, Recover to the pre-event state, Recover better than before, and Transform (10). Ridde et al. summarize these as four hospital resilience outcomes: Collapse, Deteriorate, Recover, Improve (23).

Hospital resilience improves access (in all its five dimensions, 5As: Approachability, Acceptability, Availability, Affordability, Appropriateness) to comprehensive, high-quality, patient-centered health services (24), without pushing families into poverty (25), **ultimately advancing UHC and reducing health inequalities** (Box 1). In addition, hospital resilience also absorbs the shock of the disaster or emergency, minimizing its impact on the community (22), reducing future vulnerabilities and risks through prevention and mitigation (21), and ultimately supporting national preparedness and response efforts **toward health security**. These findings are comparable with the literature where resilient health systems improve health outcomes and advance UHC and health equity while also reducing vulnerability to public health emergencies, further playing an essential role in strengthening HEDRM (4, 26, 27). This double benefit and improved performance and outcomes during “stable” (or good) and “crisis” (or bad) times is called the “resilience dividend” (12).

Box 1 **Impacts of resilient hospitals.**

- **On UHC:**
  Improve access to health and health outcomes without pushing families into poverty.
- **On health security:**
  Decrease vulnerability to various types of emergencies and improving community resilience and sustainability.
- **On health equity:**
  Challenge imbalances of power and transforms social and structural determinants that contribute to inequalities and increased vulnerabilities.

Across the literature, there is little documented about the overall attributes of resilient hospitals with only two studies explicitly mentioning attributes: Mohtady Ali et al. noted that resilient hospitals should be “*robust,” “lexible,” and “mannerly*”in order to achieve their function of having a high level of business continuity (18). To measure hospital resilience, Zhong et al. adapted the MCEER 4R framework, introducing the attributes of *Robustness, Redundancy, Resourcefulness* and *Rapidity* (28). Conversely, literature related to attributes of health systems further reflects the diversities in frameworks, terminologies, and conceptualizations (6, 8, 11–13). Kruk et al. proposed that resilient health systems should possess five elements: awareness, diversity, self-regulation, integration, and adaptability (12). Nuzzo et al. proposed 16 attributes of resilient health systems ranging from preparedness, response, and recovery plans, interventions for the various health system building blocks, surge capacity, adapting standards of care during emergencies, and commitment to quality (11). A recent systematic review of resilient systems identified six intermediate objectives or attributes: awareness, surge capacity, collaboration and coordination, resistance, access to resources, and flexibility (8). Another study found that resilience resulted in the longer-term outcomes of sustainability, efficiency, and responsiveness (5).

#### Capacities

Most studies on hospital resilience cite Zhong et al.'s inaugural definition encompassing the resilient hospitals' ability to **resist, absorb, respond, recover and/or adapt** (18, 19, 21–23, 25, 29, 30). In more recent literature, these are condensed into three hospital resilience capacities: **absorptive, adaptive, and transformative**, in line with numerous health systems resilience research (5, 6, 8, 10, 18, 23–25, 31). Hospital resilience is predominantly conceptualized within the classical DRM cycle (otherwise abbreviated as PPRR: Prevention/Mitigation, Preparedness, Response and Recovery) (15, 18, 19). One systematic review combined the PPRR cycle with the four capacities of resilience engineering, identifying the following capacities of resilient hospitals: “Potential” (to anticipate future threats and opportunities); “Actual” (to respond to events); “Critical” (to monitor ongoing developments); and “Factual” (to learn from past failures and successes) (18).

The literature is inconsistent in both hospital and health systems regarding the nomenclature, scope of definitions, order, and overlapping of these resilience capacities within the PPRR cycle (Table 2 and Annex 4). For example, Foroughi et al. described these three-tiered resilience strategies (absorptive, adaptive, transformative) over five interrelated “resilience phases” (anticipation, preparation, response, recovery, and growth) (8). Thomas et al. described health systems resilience strategies within the four stages of the shock cycle: Preparedness, Shock onset and alert, Shock impact and management (including the capacity to absorb, adapt and transform) and finally, recovery and learning (5). Khademi Jolgehnejad et al. proposed three stages of hospital resilience: preparedness, response, and recovery/growth (22). Some studies suggest that absorptive capacity may occur in preparedness, response, and/or recovery stages; adaptive capacities can be in the response or recovery, while transformative capacities overlap the recovery and future preparedness stages, closing the cycle (5, 6, 8, 23, 25, 31). Furthermore, resilient hospitals utilize all these capacities, sometimes simultaneously, across the PPRR cycle.

**Table 2:** Capacities in systematic and scoping reviews on hospital and health systems resilience (see Annex 4 for a full table of comparative definitions).

**Article/capacities**	**Absorb**	**Adapt**	**Transform**	**Learn**
Hospital resilience				
(24)	x	x	x	
(25)	x	x	x	
(21)	x	x	x	
	x	x		x
(7)	x	x	x	
Health system resilience				
(11)	x	x		x
(6)	x	x	x	
(14)	x		x	
		x		
(5)	x	x		x
	x	x	x	

In addition to these three capacities, the COVID-19 pandemic has highlighted the importance of hospitals and health systems becoming “learning organizations” to improve their performance (3, 8, 13, 23–25, 32–37). Notably, among included studies, **the capacity of learning** is only explicitly mentioned in two studies (18, 38); however other studies alluded to “growth” or “modifications” in interventions or systems as part of the adaptative and transformative capacities.

In this light, combining the most frequently cited definitions from the empirical literature (Table 2) for practical/operational purposes, the four capacities of resilient hospitals can be summarized as: 1) Prepare and absorb, 2) Respond and adapt, 3) Recover and transform, and 4) Learn and apply (Box 2). Furthermore, it is assumed that these capacities and the PPRR stages are not static nor clearly delineated; they are dynamic and evolving processes and may overlap.

Box 2 **Capacities of hospital resilience.**

- **Absorptive:** resist, prepare, or withstand the unforeseen shock of the emergency or impact of the disaster without loss of function.
- **Adaptive:** respond or can use alternate reserves or processes to maintain essential functions and meet immediate and acute community needs (ensure continuity of efficient, safe, high-quality, and person-centered health services).
- **Transformative:** recover from the disruption rapidly and at a sensible cost and reduce vulnerability to risk and improve readiness for future emergencies.
- **Learning:** Reflect and review past actions and their effectiveness to inform future actions, question assumptions, challenge and change existing learning structures (33).

#### Components

Across the literature, this category included the most diversity, including the types and number of “components”, their groupings, the terminologies used to describe them and often differed depending on the type of hazard (Table 3 and Annex 2).

**Table 3:** Components across global guidelines, systematic and scoping reviews on hospital resilience.

**Article**	**Methods**	**Type of hazards**	**# of comp**.	**Term used**	**Components of hospital resilience**
(24)	Systematic Review	Not specified	4	Factors	1) staff, 2) infrastructure, 3) management, and 4) logistics
(21)	Systematic Review	Not specified / Disasters	2, 3	Areas, Domains/Subdomains Factors also mentioned	Two areas: (physical and social), 3 domains [structural (S), non-structural (NS), and functional/operational (F)], 13-subdomains (S: building integrity, previous threats to building safety; NS, infrastructure protection, access and physical security, critical systems, equipment and supplies; F, preparation, organization, HR, comm/info systems, logistics and finance, patient care and supportive services, evacuation, decontamination, and security)
(23)	Systematic Review	Not specified / Disasters	3	Domains/ Subdomains	Adapted from **Hospital Safety Index**, structural, non-structural, and functional domains → 27 subdomains At least 151 checklist areas or “indicators”
(25)	Systematic Review + Mixed Methods for validation	Not specified / Disasters	4	Domains	1) Hospital Safety, 2) Hospital Disaster Preparedness and Resources, 3) Continuity of Essential Medical Services, 4) Recovery and Adaptation
(26, 41, 42)		Not specified / Disasters	4 + 8	Domains/ Subdomains	1) Response Capability: a) services, b) surge capacity, c) staff and training), 2) Disaster Management: (d) command, communication, cooperation, e) all-hazard preparedness, response, and f) recovery plans), 3) Disaster Resources: (g) supplies management, and 4) Infrastructural safety: including h) safety, surveillance and network backups.
(7)	Review WHO Policy Paper	Not specified	7	Components	1) Leadership and Coordination, 2) Contingency Planning and Flexible Financing, 3) Infrastructure, Logistics, and Supplies, 4) Hospital Workforce, 5) Clinical Services and Surge Capacity, 6) Risk communication and community engagement, and 7) Information Systems
(16)	WHO Guidance	Climate-resilient	4	Elements	1) health workforce, 2) WASH and waste management, 3) energy services, and 4) infrastructure, technologies, and products
(29)	Scoping review	COVID-19	4	Dimensions	1) planning, management, and security, 2) human resources, 3) information and communication, and 4) finance
(27, 28)	Scoping review and protocol	COVID-19	10 and 3	Dimensions Categories	10-dimension framework, including: governance, intervention level, workforce, culture and social values, finance, planning and supported guidance, systems specificities, health sector management, information systems, context and security Simplified into three categories: 1) human resources, 2) management and communication, and the 3) hygiene-security planning nexus

For better organization, the authors synthesize the following six components for hospital resilience **(6S):** space, stuff, staff, systems, strategies and services. Space (including structural and non-structural elements), stuff (including supplies and logistics), and staff comprise the core of a hospital, which require **systems** to function. These **systems** bridge strategies and services and enable the operationalization of other components such as **staff, space, and stuff** throughout the various stages of HEDRM. This model includes leadership and coordination, communication and information systems, and risk communication and community engagement as part of the “**systems”** component. Across the literature (Table 3), the most commonly mentioned components include **space and staff**, followed by **systems** (referencing a variety of interventions and strategies including but not limited to surge capacity, crisis and emergency management, business continuity, and systems for leadership and coordination, communications and information, resource mobilization, and community engagement) (18, 21, 25, 31, 39, 40).

**Regarding “space**,” many articles, including the systematic reviews on hospital resilience, utilized or modified the “Hospital Safety Index”(HSI), of which 111 of 151 are related to structural (or constructive), non-structural (or infrastructural) of the physical hospital building, its critical systems, and environment, named “hard resilience” (18, 21, 30, 31, 40, 41). This WHO checklist puts a significant emphasis on the safety, structural and architectural integrity of the physical building, its critical infrastructural and alternative back-up systems (i.e., power, water and sewage, HVAC, fuel, gas, hazardous waste management, and fire protection), and the quality and functionality of its medical equipment (17). The resilience of these non-structural elements, including medical systems, equipment and supplies, is integral to hospital resilience (15, 21, 40–47). Across the literature, some studies list these non-structural elements together with the structural elements under “space”. In contrast, others link these critical systems together with “stuff” or “systems”, highlighting the interdependence and interconnectedness of these components (18, 31, 48). Further to this, articles discussing hospital safety and infrastructural engineering also mention the importance of the design and planning of hospital buildings, suggesting that physical and infrastructural stability and safety are prerequisites to resilience (38, 42, 44, 47, 49). In response to COVID-19, infrastructural agility and space re-organization, such as the ability to expand and covert hospital rooms, parking lots, and residential or teaching quarters into triage, ICU treatment, isolation, and IPC spaces, were highlighted among the chief lessons learned (3, 14, 24, 25).

While some studies include the non-structural elements as part of stuff, others focus on supplies, finances, and logistics. The availability of emergency or flexible funding to ensure swift resource mobilization of resources, logistics and supplies management is also necessary for hospital resilience (3, 15, 16, 22, 24, 25, 28, 34, 49–52). A recent study found that the lack of financial resources, limited autonomy at the facility-level, and disrupted supply chains were frequently mentioned barriers to COVID-19 response and, ultimately, hospitals' timely resilience (14). This is consistent with other evidence from resource-restrained settings (16, 46).

**Staff** was highlighted unilaterally across the literature as one of the most important components of resilient hospitals (Table 3). Notably, while many articles utilize the HSI checklist, which groups human resources under the functional or operational component, recent evidence across literature on COVID-19 separates this component, further highlighting its significance to hospital resilience (10, 15, 21–25, 28, 31). Workforce volunteerism and the ability to surge additional staff were highlighted as enabling factors for hospital resilience (14, 25, 34, 35, 48, 53, 54). Notably, studies also highlighted the importance of multi-disciplinary teams for hospitals' HEDRM (3, 15, 21, 25, 50). When considering the resilience of human resources, in addition to availability, distribution, and safety, it is crucial to consider their ‘emotional capacities (e.g., empathy, motivation, and stress management); cognitive capacities (e.g., creativity, leadership and decision-making) and finally, their “epistemic” capacities (e.g., knowledge or technical competencies)' (55). The psychological resilience of the hospital workforce, their satisfaction and motivation, attitude toward the disaster and the hospitals' preparedness and response are identified as critical hospital resilience indicators (3, 31, 35, 46, 51, 56).

The interplay between the hospital's physical building (**space**) and its users (whether **staff** or patients) is at the core of the hospital's performance, functionality, and, ultimately, resilience (18, 38, 48, 51). Moreso, one study highlighted the critical combination of both hard resilience (structural and non-structural) and soft resilience (functional) to enhance hospitals' absorptive, adaptive and transformative capacities (31). This requires coordinated **planning and management** to ensure the continuous and safe delivery of essential **services**, especially during surge capacity during emergencies and disasters.

While many studies agree that hospital resilience is dependent on **planning**, studies vastly differ in defining the **systems'** component (14, 15, 18, 21, 28, 34, 41, 46, 57). Across the literature, this component widely encompasses various elements such as planning for preparedness, response and recovery, strategies and protocols, leadership and coordination, communication and information systems (Table 3/Annex 2). In other cases, this component also includes services delivery, logistics, operations, resource mobilization, and community engagement. For example, the widely used and adapted HSI checklist groups together disaster management and planning, communication, coordination, HR, logistics and finance, patient care and emergency or disaster health services, security, and infection control, under ONE domain “functional or operational or administrative” (18, 21, 41).

Hospital resilience further requires strengthening “soft resilience” through these internal assessment, planning, management, and operational systems. A recent study of hospitals' experience combatting COVID-19 found that leadership and coordination were among the most cited and critical dimensions influencing preparedness, response, and recovery (58). Strengthening the managerial competencies of hospital leaders in the face of outbreaks such as COVID-19 is found to be among the most critical lessons learned from the pandemic (59). This is similar to studies from Iran, India and the Philippines, where strong leadership enabled hospital preparedness and resilience (34, 46, 60). In addition to this, the pandemic further highlighted the importance of coordination with other hospitals, local authorities, and disaster management agencies, functional information systems (which adapt new technologies), and internal and external communication with the community, media, civic organizations, and local authority, particularly for risk communication and community engagement (3, 15). One study confirmed this, citing the lack of central incident management structures and systems as a leading factor in inefficiencies and delays in resource mobilization and operations (34). Another systematic review confirmed that the lack of a team-based approach in implementing disaster plans resulted in lower hospital resilience (18). Furthermore, strengthening hospital information systems is critical to improving resilience, allowing swifter data-driven decisions and transparent and timely communication with relevant stakeholders (15, 22, 34, 35, 40, 45, 52). Finally, constant communication and planning, along with engagement and support of local communities, is also critical to hospital resilience and is an area that requires further study (34, 52, 61).

Resilient hospitals also require **strategies** for preventing and mitigating hazards, reducing vulnerabilities and assessing overall disaster risks with proactive all-hazard PPRR plans (10, 15, 18, 28, 53, 62). One systematic review evaluated resilience as the performance gap between strategies (work as imagined) and services or operations more broadly (work as done) (13). Further, specific standard operating procedures (SOPs) related to various operations are needed to implement these strategies to ensure business continuity, functionality, and critical operations (22, 38, 45, 46, 57).

According to the WHO EMRO's Regional framework for action for the hospital sector in the Eastern Mediterranean Region, hospitals achieve their primary function when they provide efficient and equitable delivery of quality services, organized around community needs. Within this frame, best performing hospitals adapt to continue delivering high-quality and people-centered health services adapting to various challenges including but not limited to disasters, changing context, health system shortcomings and internal hospital deficiencies which they face. Furthermore, **resilient hospitals ensure the delivery of integrated people-centered health services (IPCHS)**.

Moreso, hospital resilience is highly related to community resilience and contribute to the social, physical economic, environmental, and public services (45). Resilient hospitals contribute to building stronger health systems, healthy communities, and sustainable development. Hospital, health systems, and community resilience are interdependent on one another (9, 10, 13, 15, 19, 52, 63).

The availability, functionality, agility, and resilience of these hospital components are interconnected and interdependent (48). Some scholars propose that hospitals are as resilient as their parts, including the physical (space, stuff/supplies, and staff) and operational (services, systems, and strategies) (7, 26, 43). The interdependence of these components affects hospital resilience whereby the resilience of the parts (components) contributes to the resilience of the whole (overall hospital resilience) (43). For instance, maintaining hospital functionality in crises depends first and foremost on ensuring physical (constructive and infrastructural) resilience, which allows operations and continuity of critical and essential services. Additionally, in response to COVID-19, resilient hospitals depended on the resilience of their staff (10, 15, 21–25, 28, 31). This is consistent at the macro-level, where strengthening the health systems resilience requires strengthening the resilience of the respective health systems building blocks (7, 11, 26).

### Operationalization

**Figure 5:**
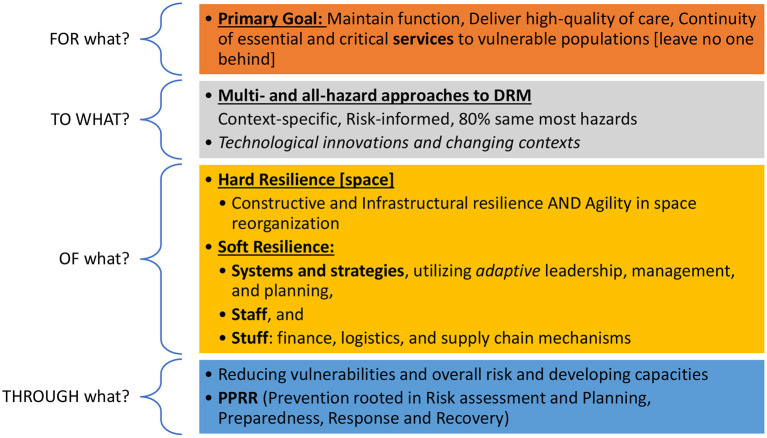
Operationalizing hospital resilience, adapted from Wiig et al.

Operationalizing resilience is one of the most difficult challenges, given the vast differences and flexibilities in definitions. To organize this section, we will follow the Resilience in Healthcare (RiH)'s framework due to its simplicity, organization, and flexibility to accommodate the application of diverse concepts and methods (55). In this framework, Wiig et al. used four core questions to guide operationalizing resilience in healthcare research: 1) Resilience FOR WHAT? (Purpose), 2) TO WHAT? (Types of disasters and emergencies), 3) OF WHAT? (Components), and 4) THROUGH WHAT? (Which capacities are applied when?) (Figure 5). The first and third questions have been answered in the conceptualization section above by defining the outcome of resilient hospitals (resilient for what) as well as their different components (resilience of what). This section will focus on answering the second and fourth questions.

Ideally, through strengthening hospital resilience, hospitals not only improve their capacities to absorb, adapt, transform, and learn over time but also their abilities to reduce risks (10, 27). Across the literature, studies predominately focus on the preparedness and response stages, with few mentioning the recovery and prevention stages (22). This is consistent with findings on health systems resilience, where most interventions and available literature focus on preparedness and response (5). One scoping review on hospital resilience to COVID-19 found that most included articles focused on preparedness and response, highlighting the capacities of healthcare facilities to *absorb* the shock by implementing short-term measures *to adapt and respond* (25). In retrospect, given the prolonged and ongoing responses to the pandemic, it is likely that responses to COVID-19 spanned the response and recovery stages (1, 2, 24). Furthermore, it is important to note that these stages are not rigid but rather overlapping and continuous (e.g., early recovery and response stages), making it difficult to separate some interventions across these stages.

Hospitals should ideally be resilient to all-hazards through each of the PPRR stages (15, 19, 28, 46, 57), as outlined in the following section. Moreso, the COVID-19 pandemic highlighted the need for hospitals' resilience to multiple types of hazards simultaneously. For example, many countries in the EMR face humanitarian emergencies, especially in fragile health systems where hospitals necessitate concurrent resilience to biological, technological, and societal hazards, and in some countries, also natural hazards (15). This requires proactive planning and prompt implementation (1, 3, 18, 57, 64). Notably, recent guidance suggests that 80% of emergency and disaster risk management is the same for most hazards, with only 20% of interventions unique to specific hazards (64).

#### Prevention rooted in risk assessment and planning

The first step of HEDRM and hospital resilience is **risk assessment** (27, 57, 64). Risk assessment includes identifying hazards, exposures, vulnerabilities (at the facility and community levels), along with capacity assessment and impact analysis (10).

Risk analysis is a critical tool and the first step toward *awareness* which has been described as a key attribute and competency of resilience (6, 8). According to one systematic review, awareness, or the “cognitive capacity” comprises the ability to detect, interpret, predict, monitor, and communicate “shocks” (8); another systematic review described this capacity using concepts in resilience engineering as “potential,” “critical” and “factual” capacities (18).

Building on emergency and disaster risk assessment, hospitals must prioritize their risks and plan accordingly. Planning is essential to HEDRM and is underscored across most studies according to multiple systematic reviews on hospital resilience (18, 21, 22). Confirming this, resilient hospitals utilize proactive and pre-emptive planning to mitigate unexpected disruptions, as embedded in resilience engineering principles of anticipating, responding, monitoring, and learning (43). As part of risk-reduction, resilient hospitals should have emergency and disaster risk reduction plans, all-hazard and hazard-specific emergency preparedness and response plans and/or programs, and recovery plans (64). In Abbasabadi Arab et al.'s model, during the prevention stage, hospitals utilize the HSI checklist to assess vulnerabilities of their structural, non-structural, and functional safety (57). Notably, the majority of literature and guidelines focused on hospital preparedness, and many studies include prevention (risk reduction and mitigation) as part of the preparedness stage (22, 65–68).

#### Preparedness

Across the literature, hospital preparedness is highlighted as the most essential stage of the DRM cycle (19, 22, 34, 35, 41, 46, 52, 60). One systematic review on hospital resilience found that more than half of included studies mentioned hospital planning and preparedness, indicating the bias the literature toward preparedness as a stepping-stone to resilience (22). A systematic review on hospital preparedness found 13 unique definitions of disaster preparedness in hospitals and 22 different operationalizations of the concept (67), further confirming the diversities in its application. Numerous systematic reviews have been published outlining comprehensive recommendations on hospital preparedness measures in case of biological hazards (65, 69), chemical incidents (66), mass casuality incidents and disasters (68), and infectious disease outbreaks such as COVID-19 (70). Despite the importance of preparedness within HEDRM, in one review of the EMR, 70% of included articles (most on all types of disasters and hazards) described hospital preparedness as very poor, poor or moderate (71). On the other hand, a recent regional study of hospitals responding to COVID-19 revealed that proactive and all-hazards preparedness at the facility level was among the chief lessons learned in building hospital resilience (3).

During the preparedness stage, hospitals are expected to provide predictions and awareness regarding the magnitude of the disruptions (22). Abbasabadi Arab et al. described the preparedness stage to include both the knowledge and capacities to anticipate, respond, and improve likely risks and their effects collaboratively and effectively (57). This encompasses interventions related to early warning systems, surge capacity, response strategies, training and disaster exercises. Another study found that preparedness required adequate and applied knowledge of the hospital resources, facilities and disaster handling processes, which ultimately improved resilience to emergencies and disasters (46). Confirming this, another review found that among the chief activities needed in preparedness is continuous learning and implementation of practical simulations exercises to familiarize front-liners as they improve knowledge of emergency activities, policies and procedures, overall competence, and confidence (72). Moreover, closing the knowledge-action gap in preparedness is of critical importance, as studies found that while preparedness plans may have been developed, the challenge arose in their implementation (13, 14, 18, 31, 34, 35, 52).

Some scholars suggest that the preparedness stage is foundational to resilience. On the other hand, one study counters this conclusion by offering an interesting perspective: “*Hospitals can be resilient even if they are unprepared and can be prepared but not resilient”* (35). Arguably, examples from the EMR show that hospitals became resilient to COVID-19 despite their lack of preparedness; through evaluating and learning from their responses to their early waves of COVID-19, they become better prepared to respond to subsequent waves and plan for recovery (3). On the other hand, the intensity, speed of onset, coverage, and duration of other hazards (e.g., drought or some infectious diseases) may allow time for hospitals and communities to learn and adapt their preparedness and response strategies during slow onset and long duration. Furthermore, preparedness is important as it underpins hospitals' response, recovery, and ultimately resilience to subsequent shocks (15, 22, 31, 41, 52).

#### Response

In the response stage, hospitals *adapt* to maintain operations and functionality. Studies suggest that response activities should be multifaceted in utilizing the various components in a manner characterized by agility, flexibility, rapidity and adaptability of the immediate and ongoing activities and operations, especially in responding to surges (34, 42, 48, 54, 57, 73).

Across studies, the most commonly mentioned components in the response stage included space, staff and stuff (40, 45). **Swift mobilization of resources** (human, material, and financial) is necessary to advance surge capacity and improve hospital operations in the response stage (22). Studies from resource-restrained and emergency settings highlighted the chronic and complex challenges in resource mobilization, citing critical shortages of health workers, emergency care and ICU specialists, cash, supplies, fuel, and frequent electricity outages impeding hospital emergency response (15, 16, 34, 42, 43). Overcoming these challenges rapidly and innovatively enabled hospital resilience through partnerships and collaborations (34, 37).

Across recent literature on hospitals' responses and resilience to COVID-19, hospitals applied multi-pronged interventions across the various components, including but were not limited to: utilizing flexible working arrangements to promote health worker well-being, regularly training and communicating with staff, optimizing the use of telemedicine e-health for services delivery, reorganizing hospital space to expand capacity for critical care and ensure safety, distributing PPEs using a risk-stratified approach, and enforcing protection protocols (24, 25, 50, 52). This is consistent with a recent regional review of hospital responses to COVID-19 in the EMR, where hospitals implemented a variety of evolving and adapting interventions (3, 14). This is further confirmed across studies on natural hazards. For instance, in the face of earthquakes, several studies highlighted hospitals' **rapid adaptive capacity** in increasing staffing to accommodate surges, redistributing, referring, and transferring patients to other facilities (40, 48). Moreover, one study described the ability to change in response to shocks or everyday challenges as an attribute of resilience, termed **surge** capacity, or “behavioral capacity” (8). Further to this, another study highlighted that the ability of the hospital network to offer redundancies in services after an earthquake resulted in a 12% increase in the hospital's resilience (calculated by recovery of loss of function or loss of services, Annex 5) (40).

This rapid adaptive capacity is essential not only for the response but also for preparedness and recovery. In cases where the response has become prolonged, such as COVID-19, a hospital's resilience is marked not only by its capacity to adapt but also by its “redundancy” or “sustainability” in managing, duplicating, or mobilizing alternative resources. For example, in the case of COVID-19, nowadays, the resilience of hospitals depends on staff burnout, running-down of oxygen supplies, and financial management and recovery of suspended elective services (24, 25, 50, 52).

#### Recovery

Hospital resilience also occurs through the **recovery stage**; however, little is documented about the activities or processes required to operationalize hospital resilience in this stage. Recovery consists of the restoration, reconstruction and improvement of facilities, livelihoods and living conditions of affected communities (57, 64). Notably, the response and recovery stages are closely interconnected, with some health systems researchers highlighting the early recovery stage overlapping between response and recovery (74). In moving from response to development through the building back better approach, hospitals should focus on early recovery (through the continuation of critical and essential services), medium-term recovery (through the restoration of disrupted health services), and finally, long-term recovery (through reconstruction, rehabilitation, and building community resilience) (64, 74).

Recovery interventions require pro-active, comprehensive, multi-stakeholder, and community-based recovery planning, damage, needs, loss and capacity assessments, and continuous monitoring and evaluation (64). Systematic, collaborative, and coordinated approaches are essential to hospitals' resilience, especially in the recovery stage (15, 18). Continuous training, teamwork, institutional learning, and adapting new technologies are key enabling factors to sustainable and effective response and recovery and, ultimately, hospitals' resilience (8, 18, 25, 32, 57, 63). Another study found that the most common adaptive strategies included empowering local leaders, increasing community awareness through communication and engagement, and enhancing community engagement (75). Moreover, strengthening leadership and coordination, partnerships, communication and information systems is essential to restoring services and trust in the community (15, 21). Resilient hospitals benefit from utilizing social capital, networks, and media (34).

In responding to natural, technological, societal hazards, hospitals should be prepared to mobilize large investments for repairs, rehabilitation, retrofitting, and reconstruction to mitigate structural and non-structural failures (21). Studies highlight the importance of constructive resilience, especially in the recovery, noting that most damages are related to inappropriate site selection for the building, lack of proper design or insufficient maintenance (21, 63). Following natural hazards such as earthquakes, the damages caused to utility networks and non-structural components had the most significant effect on hospitals' functionality and, therefore, resilience; these were also the costliest to repair and required prioritization in the recovery (21, 40, 44, 45). Studies further highlighted the need to improve the hospital buildings, engineering, and built environments to enable rapid recovery (38, 42, 45). In addition to infrastructural recovery, hospitals must also work to improve their energy, water, sanitation, hygiene, and waste management systems to improve environmental sustainability (10).

### Evaluation

Ideally, evaluation and measurement systems should span the Donabedian categories of 1) structure (or components), 2) process (or capacities), and 3) outcome (28). Nevertheless, when exploring the evaluation strategies across the literature, few reflect measures across all three categories (Annex 5). In some cases, depending on the conceptualization of hospital resilience, some indicators or evaluation measures may fit under multiple Donabedian categories. For example, one evaluation model broadly proposed three categories of Key Performance Indicators (KPIs): organizational, staff, and society results, which depending on the specific indicator, may be related to outcomes, processes or structures (57).

Overall, few validated measurement tools are proposed to assess or evaluate hospital resilience (18, 19, 21, 28). This is consistent with studies on health systems resilience which confirm the insufficient and inconsistent evidence about which aspects of resilience frameworks should be measured and which of them are measurable (6, 7). Further to this, one systematic review on evaluating hospital resilience confirmed the imbalance of literature on evaluations and tools across the PPRR cycle, citing preparedness as the most important phase of the resilience process and highlighting the limited tools for the response and recovery phases (22).

Across the literature, a variety of qualitative and quantitative measures are proposed to measure hospital resilience (18, 21, 45, 75). One systematic review revealed that most methods used to study resilience are qualitative (13). Another review found that qualitative studies captured resilience concepts more comprehensively than quantitative ones, which were limited by the paucity of available resilience data and indicators (6). Another study on resilience measurement tools found that only 18% of included articles created a quantitative resilience index and revealed that only 10% of included articles utilized validated measures (75). We need a sentence here mentioning that the below section will provide information regarding the evaluation/measurement of outcomes, capacities and components.

Regarding outcomes, hospital performance and quality are used to evaluate resilience. In evaluating hospital resilience, a systematic review found that time-based measures were among the most common strategies; measuring the quality of care through waiting time (as process indicators) was directly correlated to hospital resilience (18). Studies measured quality predominantly through patient satisfaction and waiting time; emergency department overcrowding was also noted (18, 43–45, 51, 63, 76). According to Ramandi and Kashani, the quality of hospital performance impacts waiting time and the number of deaths; this study measured resilience based on cumulative mortality (76). Hospital admissions were also used to evaluate hospital resilience, with some studies comparing this routinely collected indicator at various points in the response and recovery stages (48, 51). A systematic review of evaluation methods highlights hospital safety and functionality as critical resilience measures (18), with the complete loss of services and reduction in critical and lifesaving clinical and support services being critical indicators (40, 44, 45).

Other studies measured hospitals' performance and ultimately resilience through other routinely available data such as overall bed capacity, ICU beds available per catchment population, number of deaths (34, 40, 41, 44, 49, 51, 52, 62, 76). Additionally, resilient hospitals should also work to improve coverage and accessibility; only one article measured “leaving no one behind” through unmet demand (and waiting times) (39).

With regards to evaluating capacities, the adaptive capacity is most frequently referenced in the literature on hospital resilience (21, 29, 34, 36, 38, 43, 48, 50, 77, 78). This may be due to the wide encompassing definitions and variation of “adaptivity” [e.g., “flexibility” (8, 18, 35, 47, 48, 50, 62), “agility” (38, 44), and “performance variability” (13)] and its measures. Studying the wider literature on health systems resilience, a systematic review found the highest number of indicators across different levels of data collection was for the “absorption” domain. This study found only three indicators used for the “adaptation” domain, collected at national and organizational (including staff and patient/population) levels; in contrast, no indicators were identified for the “transformation” domain (6).

Zhong et al. were the first to propose a measurement strategy to assess hospitals' resilience using a community-centered and all hazards approach; adapting MCEER's 4R framework, the measures of robustness and rapidity are described as “ends,” whereas the redundancy and resourcefulness are described as “means.” Zhong et al. highlighted the importance of hospitals' adaptive capacity, especially the adaptive flexibility (termed: Resourcefulness) along with the ability to create or substitute services, facilities, and resources to maintain functionality (termed: Redundancy) as the means to hospital resilience (28). These two measures are combined by Fallah-Aliabadi et al. termed “infrastructural resilience” or the capability for mobilizing alternative external -human and material- resources to maintain functionality (21). Studies confirmed that hospital resilience was highly dependent on emerging adaptations, which played a critical role in redundancy and resourcefulness, complementing existing disaster plans (30, 41, 43, 48). Yin et al. further highlighted that effective adaptation is an essential attribute of hospital resilience, which requires resource mobilization or substitution, proactivity, and timely and decentralized decision-making (43). Moreso, the same study concluded that improvisation was a critical measure of adaptive effectiveness and hospital resilience. The Resilience Analysis Grid (RAG) for organizational resilience was utilized to qualitatively evaluate how the hospital responds, monitors, learns, and anticipates changing situations, comparing “work-as-done” and “work-as-imagined” (43). This measure was among the most frequently utilized in two other reviews, which measured health systems (including hospitals) resilience (Biddle and RHC). In one systematic review, the Concepts for Applying Resilience Engineering (CARE), Functional Resonance Analysis Method (FRAM), Resilience Markers Frameworks (RMF) were included, along with mostly qualitative measures, among the methods for measuring performance variability and ability to adapt in resilient healthcare systems (13). In another study, adaptive capacity explored hospitals' business continuity and operational management (18). Paterson et al. included adaptive capacity as one of three areas assessed when evaluating hospitals' resilience to climate change, while Goncalves et al. set “adaptive capacity” as one of the two primary domains assessed when evaluating healthcare organizations' resilience (77, 78).

Zhong et al.'s framework was adopted by several studies exploring hospital resilience, especially in the contexts of natural hazards such as earthquakes (21, 30, 41, 42, 48). For instance, one study that investigated hospital infrastructure resilience to natural hazards included (i) robustness through building codes and structure, architecture, planning and zoning; (ii) redundancy through planning and operations; and (iii) rapidity through communication, movement, and risk assessment (42). Expounding on this framework, the measures of rapidity and robustness will be further explored.

Across the literature, particularly articles related to natural hazards, hospitals' hard resilience is of critical importance, especially to the hospital's absorptive capacities (31, 39, 40). This inherent strength to withstand the consequences of a shock is termed “robustness” (18, 28, 30, 41, 42), the attribute of resistance (8, 40), and “constructive resilience” (21). Most proposed measures for robustness are related to hospital safety using the HSI or modified versions (21). One study utilized a customized five-dimensional City Resilience Profiling Tool (63), while another evaluated the hospital's-built environment and plans (38) to measure hospital resilience within its environment. Other studies related to natural hazards measured environmental characteristics (e.g., seismic, climate-related exposures) (10, 40), infrastructural planning and design (47, 49, 50, 63), and modeling to measure hospital's loss of function (39, 44).

Measuring hospital's **rapidity** in response and recovery is among the most important measures of hospital resilience across the absorptive, adaptive, transformative, and learning capacities (21, 53). In addition to the time-based measures mentioned above, one of the most frequently mentioned hospital resilience measurements is the **fault-tree analysis**, used to assess the **hospital's functionality** (18, 40, 45). Several studies measured functionality through the recovery or repair rates of critical function systems, including non-structural and medical systems, and on this basis, quantified the service, economic, and life losses (40, 44, 45). Highlighting the importance of the attribute of rapidity to hospital resilience, one study identified three stages of rapidity throughout the response, recovery, and better preparedness cycle. These included critical rapidity to address immediate needs, stabilizing rapidity until the hospital re-started routine activities, and recovery rapidity (48).

Finally, some studies also propose evaluating hospital resilience through the capacities of risk assessment and management and planning (18, 77, 78); one study even used a simple SWOT analysis to evaluate these capacities (36).

Given the variations in how studies conceptualize components of hospital resilience, there is subsequently a vast diversity in their measurements (Annexes 2 and 5). Studies emphasize measuring both hard and soft resilience, emphasizing the importance of both structural and non-structural robustness, as well as adaptability in “administrative or functional” domains (18, 21, 28, 29, 31, 40, 41, 45, 48, 53, 54). The most commonly mentioned components in evaluating resilience included: staff, structure/space, and stuff, especially in studies utilizing fault-tree analyses to measure functionality (40, 44, 45). Most studies, particularly those evaluating resilience to natural hazards, evaluate or adopt the structural, non-structural, and functional domains using the HSI checklist (18, 21, 30, 31, 40, 41, 48). In addition to “constructive resilience,” staff resilience was fundamental to hospital resilience during emergencies and disasters. Studies utilize various qualitative measures to evaluate staff satisfaction, burnout, knowledge, attitudes, and perceptions (48, 51, 78, 79).

Only one study proposed a composite hospital resilience index based on the presence or absence of 102 indicators across eight domains (34). The weighted average of each domain was calculated, factoring in the reported available number of indicators for each of the eight domains adopted from the Zhong et al.'s framework (Annex 5).

## Discussion

### Summary of the main findings

**Figure 6:**
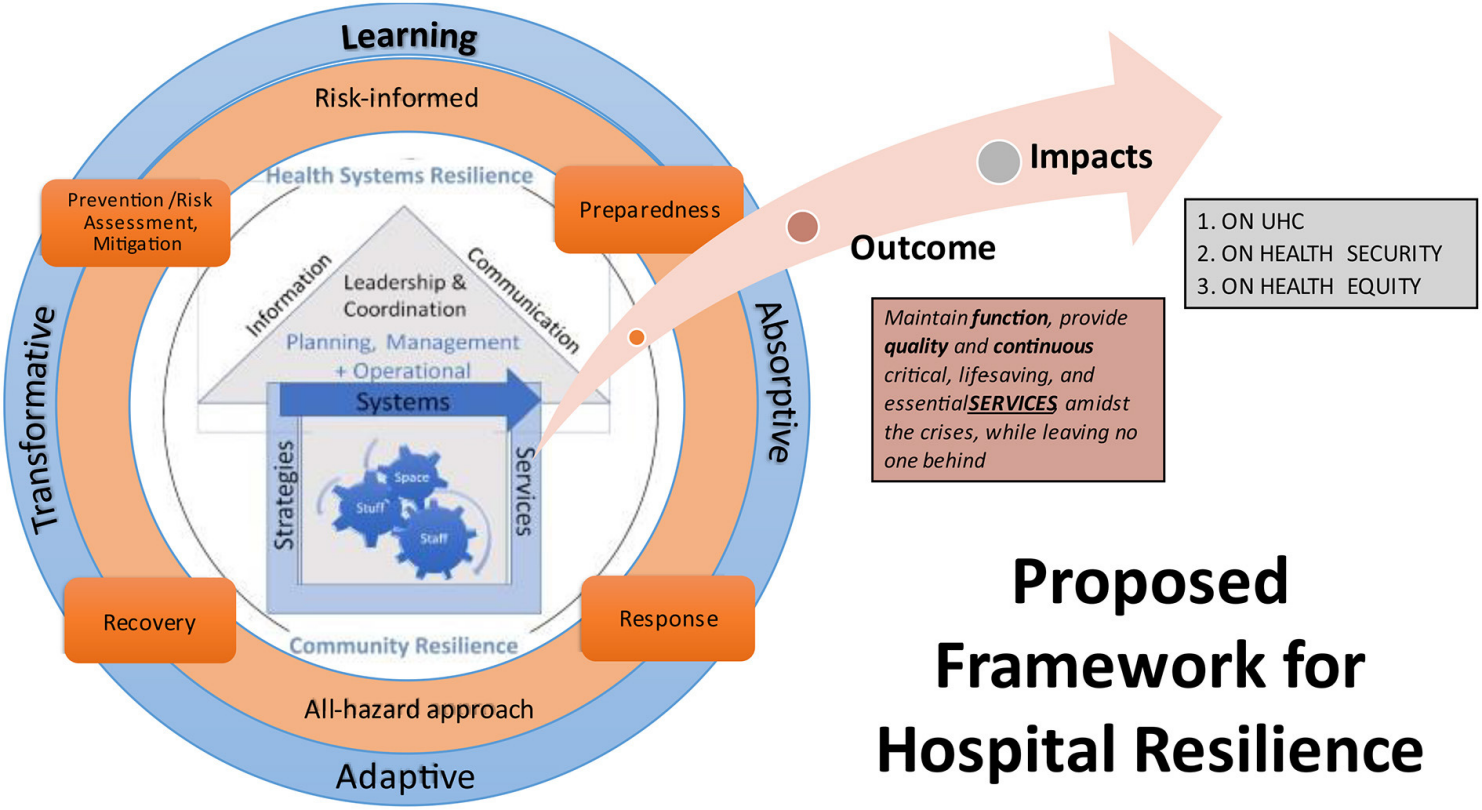
Proposed conceptual framework for hospital resilience.

This study aimed to investigate how hospital resilience is conceptualized, operationalized and evaluated in the empirical literature. The proposed conceptual framework (Figure 6) synthesized the varied evidence in the literature, offering a starting point and consensus to guide discussions on strengthening hospital resilience operationalization and evaluation. Hospital resilience is conceptualized by 6 components (6S), 4 capacities, 1 primary outcome, and 3 impacts (Table 4).

**Table 4:** Summary of proposed conceptual framework on hospital resilience.

**Components *(6S)***	**Capacities**	**Outcome**	**Impacts on**
1. Space 2. Staff 3. Stuff 4. Systems 5. Strategies 6. Services *Embedded* in health systems and community resilience	1. Absorptive 2. Adaptive 3. Transformative 4. Learning These are utilized *throughout PPRR* stages	*Maintain function, provide high-quality and continuous critical, life-saving, and essential services, amidst the crises, while leaving no one behind*	1. Universal Health Coverage 2. Health Security 3. Health Equity

The framework consists of three concentric layers (showing the components and capacities) and an arrow showing the outcome and impacts. The primary outcome of resilient hospitals is to maintain their function, which occurs when they provide high-quality (safe, effective, patient-centered, timely, efficient, equitable) and continuous critical and essential services amidst crises while leaving no one behind (15). As a result, resilient hospitals improve access and coverage and reduce risks and health inequalities, further contributing to advancing universal health coverage, improving global health security, and promoting healthier populations.

At the core (Figure 6), the framework shows a hospital with its 6 components, interconnected, and embedded within health systems and community resilience. The second layer shows that hospital resilience manifests throughout all the four stages of the HEDRM cycle (PPRR), within an risk-informed and all-hazard approach. The third layer (blue circle) shows the four resilience capacities which occur throughout the PPRR cycle. Hospital resilience manifests through absorptive, adaptive, transformative, and learning capacities. In some frameworks, these “capacities” are described as resilience “processes” (10, 23), “strategies” (8), “abilities” (11, 14), capabilities (18), or “stages” (12). Despite these differences in terminologies, order/organization, and controversial overlap within the HEDRM cycle, these capacities actuate hospital resilience, and are not static but rather dynamic, continuous processes that occur over time (9, 21, 43, 51).

Regarding the operationalization of hospital resilience, there are no conceptual frameworks to guide the application, monitoring, and evaluation of this concept into practice (22). Nevertheless, the literature suggests that an all-hazard approach should be applied as hospitals utilize multi-pronged interventions utilizing all components across each of the PPRR stages (15, 19, 28, 46, 57). Applying an all-hazard approach, hospitals only need to tailor 20% of their preparedness and response activities for specific hazards (64). The preventive aspects of resilience are critical as the first step of hospital resilience is risk assessment, including monitoring, identifying, and analyzing risks, hazards, vulnerabilities, and exposures. This informs prevention (risk-reduction or mitigation), planning and preparedness. Strengthening pro-active and comprehensive preparedness for all types of hazards is also necessary; especially as studies point to need for shifting the paradigm in toward a proactive, risk-informed, adaptive, innovative, and collaborative approach to HEDRM (3, 27, 59).

With regards to evaluation, the nuances and differences in conceptualization are ultimately reflected in the diversity of assessment and measurement strategies; the lack of measurable hospital resilience indices reflect the complexity of this concept presented in the literature. There is a need to clearly define and encode resilience measures explicitly specifying thresholds for timeliness, completeness, and comprehensiveness of evaluation frameworks (6, 18, 21, 28). Factoring the time between hospitals' exposure to shocks and their responses, resilient hospitals rapidly adapt to implement multi-level and coordinated interventions to maintain functionality and mitigate a drop in performance. Additionally, when evaluating hospital resilience, adaptability (including surge capacity), flexibility, and rapidity are critical enablers, especially within the closely interlinked response and recovery phases. This is especially the case during prolonged response phases, such as COVID-19, where stabilizing rapidity in early recovery was critical for hospital resilience (48). Moreover, the absence of pragmatic, comprehensive, and validated measures to evaluate hospital resilience is a critical evidence gap that requires timely and further research (19, 22, 24, 28).

### Implications and challenges

This study confirmed that hospital resilience is a dynamic process within complex and dynamic systems (75). To guide meaningful and effective action at facility and country level, consensus is needed, along with further research, to create practical guidance for operationalizing and evaluating hospital resilience.

Despite the growing interest in the concept of “resilience” in global health, “hospital resilience” is a new concept in the literature (18, 22). At this time, there is no comprehensive framework conceptualizing or measuring hospital resilience, while few health systems, community, or organizational resilience frameworks specifically focus on hospitals (6, 18, 21, 22, 28). Furthermore, the conceptualization of hospital resilience remains nuanced, with vast discrepancies and diversity capturing these complex definitions and terminologies (18, 21, 25, 29, 30, 53, 75). For instance, while most studies and frameworks agree regarding the capacities of resilience, they are still vaguely defined and measured in the scientific literature (48). Additionally, another challenge in comparing and synthesizing these concepts is that various frameworks focus on different conceptual dimensions (e.g., capacities vs. outcomes) (13). These ambiguities make it difficult to translate and evaluate resilience practically at the hospital level (6, 13, 19, 29, 75). The proposed conceptual framework presents a baseline to enable various actors, academics, hospital managers, and policymakers to share a common understanding and language to enable systematic action toward strengthening hospital resilience.

Overall, there is little empirical evidence on operationalizing resilience (25). A recent review found that most published resilience models, frameworks, and theories only focus on one or two dimensions of operationalization (8). Nevertheless, this study found that hospital resilience occurs throughout each of the PPRR stages utilizing the 6 components and 4 capacities. Strengthening hospital resilience requires both hard and soft resilience with specific interventions to improve hospitals' routine operations prior to shocks and emergencies, as well as their capacities for HEDRM, during and after events. Hospital resilience requires strengthening planning, managerial, and operational systems, including leadership and coordination, communication and community engagement, and information systems, which directly affect hospital's abilities to absorb, adapt, transform, and learn across the PPRR stages. Notably, one study mentioned that hospital resilience is a culture (43), reminiscent of the cycles of quality improvement and continuous learning. This is also consistent with findings exploring organizational resilience which focused on motivating and building the resilience of individuals and teams (3, 46, 51, 55, 56). Moreso, in FCRS, hospital resilience and services continuity often depended on flexible financing to ensure swift procurement, equitable distribution, and efficient use of resources (3, 15, 16, 22, 24, 25, 28, 34, 49–52). Further to this, strengthening hospital's resilience requires improving health systems and community resilience; and the reverse is symbiotic (9, 10, 13, 15, 19, 52, 63). Without resilient hospitals, health system cannot be resilient; furthermore, multi-sectoral interventions, using whole of government and whole of society approaches, are needed to strengthen resilience at the national level to mitigate the impacts of shocks on hospitals' (5, 7, 9, 23, 26, 34, 52). Further research and detailed guidance are needed to delineate the interventions, interlinkages, roles, and responsibilities, between hospitals, primary-care and other service delivery actors, health systems, non-health emergency actors, and communities throughout the process of strengthening resilience.

Few studies mention the intersections of the four ‘operational dimensions' across the “through what” and “of what” dimensions of Wiig et al.'s framework. Khademi Jolgehnejad et al.'s systematic review on hospital resilience highlighted this intersection, describing 22 “factors” across the three resilience stages of preparedness, response and recovery and four components of staff, infrastructure, management, and logistics (22). A similar matrix is presented when proposing interventions to strengthen health systems resilience across four “building blocks” of finance, governance, resources, and service delivery across the four stages of a shock, starting from preparedness to recovery (5). Olu proposes interventions in a 4-by-6 matrix whereby strengthening the resilience of each of the six building blocks throughout the four stages of the PPRR cycle contributes to health systems resilience (26). Moreso, in response to the pandemic, hospitals adapted to new technologies such as telemedicine and vaccines further transforming service delivery; as such, another paradigm of “positive resilience” should be considered not only to disasters and emergencies but also to improvements in response to changes in health systems or national contexts (56). The empirical literature has failed to integrate this resilience's dynamic aspects in its operationalization, especially at the hospital level (6, 9, 55). Therefore, operational guidance must consider these nuances and diversities in conceptualizations, and defining how, when, using what, and by whom hospital resilience should be applied and evaluated.

Given the complexity and fluidity between the PPRR stages, especially in chronic and prolonged crises, further studies are also needed to define the various hospital resilience capacities within this cycle. Additional guidance is needed to the shift toward risk-based and all-hazards approaches and ensuring hospital resilience throughout the PPRR cycle toward building back better (15, 19). Across the literature, most studies focus on hospitals preparedness, some on response, few on recovery, and fewer highlight their overlap (22). Few studies mentioned the pre-requisites or enabling factors to improve hospital resilience, and further research is needed to guide implementation at the hospital level, especially in multi-hazard prone contexts. This review included a concentration of studies related to natural hazards, with only one related to societal hazards; this is consistent with systematic reviews that indicate this focus on natural hazards in the hospital resilience literature with limited literature on managing biological or multiple hazards (18). More so, at the time of this study, there was a lack of systematic and comprehensive guidance on improving hospital resilience during or after COVID-19 (25). Operational guidance must factor hospitals' responses to numerous types of hazards simultaneously and enable hospitals to prioritize risks and interventions that bolster their preparedness, especially in FCRS.

As previously mentioned, evidence on hospital and health systems resilience in LMICs, especially in emergency settings and humanitarian conflicts, is very limited. Operational research on improving hospital resilience in these settings is timely and needed. This study confirmed the geographical and publication bias toward the Global North inhibits contextualized and effective operationalization in LMICs where arguably hospitals have historically been more resilient in managing everyday and chronic stressors (6, 13, 18, 22, 25). To bridge this gap between “routine or everyday” resilience and HEDRM-specific resilience, one study recently introduced a health systems resilience index based on Kruk et al.'s five inherent system resilience capacities and IHR emergency preparedness and response capacities (80). Further to this, a synthesis of disaster resilience measurement methods and indices highlighted that most studies are measuring resilience focus on a certain region, scale and type of disaster without deriving inferential models or equations for future use nor application at the facility-level (75). The application, evaluation, and documentation of hospital resilience in resource-restrained settings are not only necessary but immensely valuable in offering key insights and lessons in implementing resilience due to their chronic exposure to shocks and ability to continue providing essential and emergency health services (15, 37). Moreso, further research is needed to explore the links and tradeoffs between strengthening and evaluating hospital resilience within routine contexts (e.g., hospital's everyday resilience, continuous improvements to day-to-day, responses to chronic stressors) vs. acute or event based HEDRM.

Guided by this scoping review and addressing some of the gaps it exposed and the consensus of the expert consultation, a simple and easy-to-use operational matrix and guidance on strengthening hospital resilience is being developed and piloted in FCRS as part of the larger regional project. This guidance will enable hospital managers to utilize a risk-informed, step-wise approach to strengthen their hospital resilience. As for evaluation, additional research is being conducted as policymakers and hospital managers alike would greatly benefit from improving the quality, availability, and validation of data and evaluation tools and frameworks (6, 28, 29, 75). Furthermore, both qualitative and quantitative evaluation tools are needed to evaluate hospital resilience comprehensively.

### Strengths and limitations

Among the first specifically exploring the concept of “hospital resilience,” this study makes a substantial contribution to health systems and DRM fields alike by synthesizing the conceptualization, operationalization, and evaluation of hospital resilience in the available empirical literature. The study findings were also present and validated by a group of international and multi-disciplinary experts during the first ever WHO expert consultation on hospital resilience who reached a consensus regarding the conceptualization and major interventions for operationalization, and evaluation. As part of the larger mixed-method study, the findings were used to inform the qualitative tools used during the in-depth key informant interviews and online survey, which were triangulated to inform the overall study findings. However, this study was not without limitations.

Due to the ongoing research on “resilience,” especially in the aftermath of COVID-19, some articles may have been missed. This review explored “hospital resilience”; as such, the search terms were geared to finding articles specifically referring to this concept. Moreover, given the broad conceptualization of what resilience is (Table 1), some studies which apply or assess aspects of hospitals resilience may have been missed if they utilized different terminologies (6). This review found that hospital resilience is embedded with both community and health systems resilience; for the purposes of this paper, the concept of hospital resilience was viewed as separate but parallel to health systems and community resilience. Nevertheless, health systems encompass hospitals and hospital workers are essential leaders and members of their communities; the specific interlinkages and role of hospitals in relation to other health services delivery and HEDRM actors must be further clarified and delineated. To counteract this and capture the relevant articles, systematic reviews on health systems resilience and relevant UN documents and guidance were reviewed and their reference lists snowballed. Notably, another limitation is the lack of universal definition of what a hospital is with varying definitions for different readers and stakeholders. Additionally, resilience was historically documented using the language of “preparedness” and “crisis management,” especially in literature before 2013 (18, 22). To counteract this limitation, systematic reviews on hospital preparedness were also reviewed. This study confirmed a geographic bias toward the Global North; with few articles specific to the African and South American Regions. To counteract this limitation, the authors reviewed all WHO guidance, including health systems resilience toolkits piloted in AFRO and PAHO's Safe Hospitals programs.

## Conclusions

Similar to health systems resilience, hospital resilience is an under-researched and nascent concept in the literature, with limited evidence to translate the theoretical into practice. These nuances in conceptualization ultimately impact how hospital resilience is applied and evaluated. This study set a baseline of available concepts and subsequently gaps in literature, allowing for a consensus among different stakeholders and exposing the opportunities for further research. Conceptualizing hospital resilience should encompass its dynamic and evolving state, its outcomes, capacities, and components, within the HEDRM cycle while utilizing an all-hazard approach. Hospital resilience, particularly in LMICs, must consider resource-limitations, multi-hazard approaches, technological advancements, and evolving health systems shocks and environmental risks. Based on this review which is part of a large mixed method study, evidence and an operational guide including relevant tools are being developed to support hospital managers in operationalizing and evaluating hospital resilience. They will be piloted in different setting including fragile and low resources settings. Further qualitative and quantitative research is needed for the operationalization and evaluation of hospital resilience comprehensively and pragmatically, especially in fragile and resource-restrained contexts.

## Data availability statement

The original contributions presented in the study are included in the article/Supplementary material, further inquiries can be directed to the corresponding author.

## Author contributions

MK: conceptualization, data collection, data analysis and interpretation, and drafting and finalization of manuscript. HR: conceptualization, data analysis and interpretation, critical revision, and finalization. DS, JA, AA, HS, and AC: conceptualization and critical revision of manuscript. All authors have read and approved the final manuscript.

## Conflict of interest

The authors declare that the research was conducted in the absence of any commercial or financial relationships that could be construed as a potential conflict of interest.

## Publisher's note

All claims expressed in this article are solely those of the authors and do not necessarily represent those of their affiliated organizations, or those of the publisher, the editors and the reviewers. Any product that may be evaluated in this article, or claim that may be made by its manufacturer, is not guaranteed or endorsed by the publisher.
