# Supplementary material for: What is “hospital resilience”? A scoping review on conceptualization, operationalization, and evaluation
Source: Front Public Health. 2022 Oct 14;10:1009400. doi: 10.3389/fpubh.2022.1009400 (PMC9614418; doi:10.3389/fpubh.2022.1009400)
Supplement: Supplementary file 1 [file Table_1.docx]

# Annex Guide:

- Annex 1: Search Strategy
- Annex 2: Extraction table (Summary of hospital dimensions and evaluations in the literature)
- Annex 3: Conceptualization extractions for systematic and scoping reviews
- Annex 4: Capacities in systematic and scoping reviews on hospital and health systems resilience
- Annex 5: Hospital resilience measurement tools across the literature

# Annexes

## Annex 1: Search Strategy

| **Search Strategy Area** | **Details** |
| --- | --- |
| Geographic Limit | No limitations |
| Species | Human |
| Language | English, Arabic, French |
| Time frame | 2011-2021 |
| **Peer-review publications** | |
| Databases | PubMed, Web of Science, IMEMR, Embase, CINAHL, Google Scholar |
| Keywords | “hospital*” or “health facilit*” AND “resilien*” |
| MeSH | (((health facilit*[Title/Abstract]) OR "hospital*"[Title/Abstract] or "hospitals"[MeSH Terms] OR "hospitalization"[MeSH Terms]) AND ("resilience"[All Fields] OR "resiliences"[All Fields] OR "resiliencies"[All Fields] OR "resiliency"[All Fields] OR "resilient"[All Fields] OR "resilients"[All Fields])) AND (humans[Filter]) AND (english[Filter]) AND (2011:2021[pdat]))) |
| **Grey literature** | Including presentations, conference papers, abstracts, blogs, policy reports, relevant publications from UN partners such as UNISDR, UNICEF, World Bank, Asian Development Bank, MSF; Global and regional academic partners (e.g. AUB, the Asian Disaster Preparedness Center (ADPC)), and Hospital specific organizations (e.g. IHF) |

## Annex 2: Summary of hospital dimensions and evaluations in the literature

| **Source** | **Type of Article** | **Country** | **Type of hazard or emergency** | **Dimensions of hospital disaster resilience** | **Number of indicators mentioned** |
| --- | --- | --- | --- | --- | --- |
| Crisitian | Commentary | N/A | Not applicable | **Structural** components (e.g. infrastructural safety), **non-structural** components (e.g. staff capability), emergency medical **functions** (e.g. critical care, on-site rescue, and surge capacity), and disaster **management** mechanisms (e.g. plans, crisis communication, and cooperation) | Not applicable |
| Fallah A. et al. | Systematic Review | N/A | “Disasters” | Constructive, Infrastructural, Administrative (mirroring HSI) | 129 |
| Gupta et al. | Mixed Methods | India | Hydrological disasters (floods) | i) Healthcare **infrastructure** planning | 21 |
|  |  |  |  | ii) design and **planning** of hospital buildings |  |
|  |  |  |  | iii) emergency **service** and management. |  |
| Mani et al. | Commentary | Indonesia | “Disasters associated with natural hazards” | Safe **buildings**, | Not applicable |
|  |  |  |  | Making existing structures disaster resilient (through **assessments and risk reduction activities),** and |  |
|  |  |  |  | Developing functional, fool-proof **emergency response plans** |  |
| Chand and Loosemore | Review | Australia | Climate/ Extreme weather events | 4 areas: 1) **Building** (Building, window, fire alarm, lifts, stairs, roof access, door, room, façade, structure/structural damage, water, power/electricity, generator, light, air conditioning, phone line/telecom, sewerage, equipment) | N/A |
|  |  |  |  | 2) **Environment** (Corridor, ventilation, heat, cold, humidity, lighting, air quality, temperature, air flow, smoke, infection control) |  |
|  |  |  |  | 3) **Activity** (Training, drill, staff, evacuation, transport, maintenance, repair communication, retrofit) |  |
|  |  |  |  | 4) **Objective** (Plan/planning, debriefing, service continuity, business continuity, preparedness, patient treatment, emergency supplies |  |
| Mohtady Ali et al. | Systematic Review | N/A | “Disasters” | 2 areas: (**physical and social**), 3 domains (HSI : structural, non-structural, and functional/operational) | 13-subdomains |
| Samsuddin et al. | Qualitative | Malaysia | “Disasters” | HSI: Structural, non-structural, functional | 243 preparedness attributes and 23 resilience indicators |
| Takim et al. | Mixed Methods | Malaysia | “Disasters” | HSI: Structural, non-structural, functional | 48 out of 129 items are regarded as the utmost critical |
| Zhong et al. 2013 | Systematic Review | N/A | *All hazards* | 4 Areas: Emergency Medicine Response Capability, Disaster Management Mechanisms, Disaster Resources, and Hospital Infrastructural Safety | 4 Rs to measure resilience; 23 indicators |
| Zhong et al. (all 3 articles from 2014) | Systematic literature review and Modified-Delphi consultation | China | “Disasters” | Eight key domains were identified: hospital safety, command, communication and cooperation system, disaster plan, resource stockpile, staff capability, disaster training and drills, emergency services and surge capability, and recovery and adaptation. | 4 Factors, 8 Domains, 43 Key indicators |
| WHO Climate Resilient Health Facilities | Report | N/A | Climate Resilience | 4 components: health workforce, WASH and waste management, energy services, and infrastructure, technologies and products. |  |
| Goncalves et al. (2019) | Quantitative | Spain | All hazards | Planning and adaptive capacity | Shortform Benchmark resilience tool for healthcare organizations: 2 domains, 13 areas |
| Paterson et al. (2014) | Quantitative | Canada | Climate Change | Health care facilities will need to **assess climate change risks** and adopt **adaptive management** strategies to be resilient | 4 areas (general info, assessing climate-related risks, risk management, building capacity), 82 indicators |
| Jacques et al (2014) | Case Study | New Zealand | Earthquake | The hospital’s ability to bounce back from a disaster (or its resilience) and manage hospital surge while maintaining healthcare delivery to its community. Adapting HSI, includes structural, non-structural, functional (service redistribution and capacity building strategies) | Qualitative measures for functionality, Y indicates a loss of service, where R indicates a reduction in service, and N indicates no loss of service; Quantitative: Number of hospital beds and staff |
| Rahmani et al (2021) | Case Study | Iran | Earthquake | Urban resilience: The components or attributes and their criteria include: 1) Physical Attributes (Site plan, Hard infrastructure, Built-up environment), 2) Functional Attributes (land-use zoning, socio-economic, transportation), 3) Spatial attributes (accessibility and development plans), 4) Organizational attributes (institutional structure, hospital level structure, hospital functional scale), and 5) Functional evolution (role changing). Each of these has specific indicators. | 5 components, 12 criteria, and 36 indicators |
| Moitinho de Almeida (2021) | Mixed Methods | Nepal | Earthquake | Adaptive flexibility/ Adaptive capacity highlighted. Hospital resilience was explored through exploring hospital admission (length of stay) as well as staff attitudes and behaviors. It highly dependent on emerging adaptations, pre-existing plans, staff resilience. | N/A |
| Shang et al (2020) | Mixed Methods | China | Earthquake | Resilience demand is expressed as desirable recovery time of hospital system after earthquakes. Seismic resilience is quantified based on probabilistic seismic fragility analysis. The seismic resilience of an engineering system is often assessed using three indices, i.e., the recovery time, the economic loss, and the casualties. | 7 critical hospital sub-systems, 38 components |
| Hassan and Mahmoud (2019) | Quantitative | USA | Earthquake | Hospital resilience as defined by hospital functionality and estimating recovery. Hospital functionality is defined in relation to **losses** to the structural components, non-structural components, and systems/contents, which depends on the predicted damage resulting from the scenario event and can be calculated using fragility functions. Measuring resilience (area under recovery curve) through using fault tree analysis and modelling functions. | Adapted from Jaques et al, 3 hospital functionality domains (availability of staff, space and supplies) and 30 factors |
| Boeriu et al (2016) | Qualitative | Romania | Mass Casualty | Resilience defines the reserve capacity of the system in the disasters or mass casualty incidents, which requires far superior or different resources to those current, on extended periods, in order to resolve the crisis. | N/A |
| Capolongo et al (2020) | Qualitative | Italy | COVID-19 | The strategies are organized into two tiers: I) design and II) operations. The (I) Design phase strategies are: 1) Strategic Site Location; 2) Typology Configuration; 3) Flexibility; 4) Functional program; 5) User-centeredness. The (II) Operation phase strategies are: 6) Healthcare network on the territory; 7) Patient safety; 8) HVAC and indoor air quality; 9) Innovative finishing materials and furniture; 10) Healthcare digital innovation. | N/A |
| Abbasabadi Arab et al (2019) | Mixed Methods | Iran | All hazards | The Hospital Disaster Risk Management Evaluation (HDRME) consists of 8 constructs, including 7 enablers (management and leadership; risk assessment; planning; prevention and mitigation; preparedness; response, and recovery) and one result (key performance results). These constructs were further broken into 27 sub-constructs. Management and Leadership (3), Risk Assessment (2), Prevention and Mitigation (3), Preparedness (3), Response (6), Recovery (4), KPI Results (3) | 27 |
| Ridde et al (2021) | Protocol | Brazil, Canada, China, France, Japan, and Mali. | COVID-19 | 10 Dimensions of hospital resilience (governance, intervention level, workforce, culture and social values, finance, planning and supported guidance, systems specificities, health sector management, information systems, context and security). These are simplified into three categories: 1) human resources, 2) management and communication, and the 3) hygiene-security planning nexus/ 3 Hospital Resilience Processes: Absorption, Adaptation, Transformation 4 Hospital Resilience Outcomes: Collapse, Deteriorate, Recover, Improve/ 5 Impacts on Health Access: 5As Approachability, Acceptability, Availability, Affordability, Appropriateness. | National and facility-level checklist protocol |
| Labarda et al (2017) | Mixed Methods | Phillipines | Typoon | Hospital resilience depends on decreased vulnerability to the shocks brought by disasters and increased adaptive capacity brought by improved choices and opportunities. Hospital resilience index, derived from a self-reported checklist (Yes/No), adapted from Zhong et al. 2014. | 8 Domains from Zhong et al, 2015; and 102 checklist indicators |
| Samsuddin et al. (2017) | Qualitative | Malaysia | “Disasters” | Disaster resilience combines hard resilience (structural and non-structural) and soft resilience (functional). Both hard and soft resilience strategies will result in enhancing the hospitals’ absorptive, adaptive and restorative capacity. | N/A |
| Romani et al (2021) | Mixed Methods | Italy | COVID-19 | Hospital's continuity of essential and critical services, reducing infectious rates. Most important factors include: Adapting new technologies, strengthening community-hospital partnerships, data-based and swift decision-making, Organizational preparedness and response plans, Safety of staff | N/A |
| Barbash and Kahn (2021) | Commentary | N/A | COVID-19 | Organizational resilience characterizes firms that rapidly adapt in response to an existential challenge, enabling both an attenuated effect from the challenge and a more rapid recovery; it is the flexibility to pivot as new and unexpected challenges arise, and to absorb unexpected shocks that cannot be avoided even with proper planning. Resilient hospitals have more degrees of freedom, allowing them to consider a range of solutions to each problem and quickly pivot when a preplanned strategy is not working. The hospitals could then rapidly and effectively implement novel solutions instead of simply relying on preplanned solutions that might not fit the current problem. Resilient hospitals 1) respond to surges while ensuring a high-quality of care, 2) coordinate with local actors, regional hospitals, etc to rapidly and safely transfer cases, 3) reduce cross-infections and accommodating urgent needs of non-COVID patients, 4) preserve access to the community, especially the vulnerable, 5) protect wellbeing of frontliners | N/A |
| Yin et al (2020) | Qualitative | Singapore | COVID-19 | Adapted from common themes of resilience engineering, resilience is 1) being proactive and pre-emptive (staying ahead of the curve, to anticipate and withstand varying and increasing loads), 2) requires resources (or the capacity to generate or mobilize alternatives) and effective adaptation (which requires access to space, equipment, manpower, and expertise), 3) is timely, decentralized decision-making, 4) involves managing trade-offs (efficiency, thoroughness, trade-offs, safety, productivity, economic gains), 5) is cultural. | Resilience Analysis Grid: 4 components |
| Hansapinyo (2018) | Quantitative | Thailand | Earthquake | Lifeline systems: 1. Electricity, 2. Air Condition, 3. Communication, 4. Water Supply, 5. Stream, 6. Medical Gas and 7. Water treatment | Survey using Likert scale |
| Ramandi and Kashani (2018) | Quantitative | N/A | Earthquake | Hospital disaster resilience is the ability of the hospital to respond, resist and absorb the disaster effects to provide health care, and then return to the basic or acceptable level of service. Proposing Hospital Network Resilience Index (HNRI). Two of the most important components of the research are hospital accessibility and capacity. They influence patients’ waiting time. Other components are intensity of the hazard, quality of buildings and population of the region. These three components affect number of injured populations. Quality of hospital performance is important in reducing waiting time and number of dead people. Resilience can be measured based on total dead number. | 7 measurements: a) Number of dead people before earthquake, b) Number of dead people after earthquake, c) Number of transferred patients before earthquake, d) Number of transferred patients after earthquake, e) Network waiting time before earthquake (min), f) Network waiting time after earthquake (min), g) Random treatment time (min). |
| Norazam | Mixed Methods | Malaysia | Flooding | Hospital’s failure to absorb or withstand pressure of disaster with a certain magnitude affects their performance and functionality. The investigation of hospital infrastructure resilience includes (i) robustness through building codes and structure, architecture, planning and zoning; (ii) redundancy through planning and operations; and (iii) rapidity through communication, movement and risk assessment. | |
| Ghanaatpisheh et al (2019) | Qualitative | Iran | Disasters and Emergencies | A resilient hospital should be able to resist, absorb, respond to, and recover from the impacts of disasters as well as continues its normal operation. | N/A |
| Ybarra (2019) | Commentary | USA | Hurricane | Organizational resilience through leadership behaviors of adaptability, empowerment, and social justice. Organizational resilience is the ability to thrive amid adversity and unpredictability.2–5 It is an active measure and not merely a passive reaction to stress. Taking a proactive stance on how adversity impacts the hospital is vital to its ability to be resilient. **Adaptability** is an indicator of resilience: Hospital's ability to proactively assess resilience and absorb variability and stress is paramount. The model of reactivity is taxing and costly in terms of emotional capital and psychological resources. The ability to quickly shift from centralized to decentralized decision-making are ahead of the curve is key. **Empowerment**: Eliciting feedback from team members on a variety of factors provides a different lens to view concerns, building resilience, as a part of everyday culture, resides in the encouragement of team members to endorse and determine the pathway for well-being. **Social justice**: Diversity enables the organization to leverage strengths from lived experiences and appreciation of socioeconomic and cultural differences. | SWOT |
| Pishnamazzadeh et al (2020) | Mixed Methods | Iran | N/A | Bounce back of a system or an entity after disaster, system capability for easy recovery after changes, maximum disruption that a system can tolerate and not be turbulent, organization capability for rapid response to unfavorable events, and system ability to reduce failures. | 4 KPIs of hospitals’ resilience considered: patient satisfaction, patient waiting time, staff burnout and staff satisfaction |
|  | Quantitative | USA | Multiple (pandemic, earthquake, Flooding, MCI) | Adaptive strategies include: patient transfer, resource sharing, capacity enhancement stratgies such as modified operations and alternative standards of care (i.e. cancellation of scheduled operations, longer shifts for staff, lower staff-to-patient ratios). | |
| Ravaghi et al. Lessons | Qualitative | Regional | COVID | 10 checklist dimensions | N/A |
| Ravaghi et al.Plos One | Qualitative | Regional | COVID | 10 checklist dimensions | N/A |
| EMRO: Policy Paper | Report | Regional | N/A | 7 dimensions | N/A |

## Annex 3: Conceptualization extractions for systematic and scoping reviews

|  | **Use these (inputs)** | **To do this (actions/activities)** | **Resulting in this (outputs/outcome/impacts)** |
| --- | --- | --- | --- |
| (15) | Adapted from the **Hospital Safety Index**, this includes 3 domains (named types of resilience): constructive, infrastructural, administrative, 27 subdomains, at least 151 relevant indicators  **Infrastructural** resilience includes utilities and services.  **Administrative** resilience includes all activities for hospital disaster management, such as **hazard and vulnerability reduction measures, preparedness, response, and recovery plans, along with all community-based activities** | **Constructive** resilience for optimizing the function of the hospital to be **inherently flexible, strong, adaptive to emergency situations.** It is associated with ***robustness*; or the ability of hospital system to withstand a given level of shocks.** | **Decreasing** vulnerability to the shocks brought by disasters  +  **Increasing** adaptive capacity brought by improved measures and opportunities  (Adapted from WHO Operational framework for building climate resilient health  systems; 2015) |
| (8,  17) | 4 main domains, 12 subdomains, and 46 indicators related to hospital’s key capacities to cope with disasters.  The domains and subdomains include:  1) hospital safety (surveillance, safety/security),  2) hospital **disaster preparedness and resources** (disaster planning and procedure, crisis communications, community, connectedness, available resources and logistics management),  3) **continuity of essential medical services** (emergency medicine, medical continuity and surge capacity),  4) recovery and adaptation (recovery, evaluation and adaptation). | Hospital resilience is the capability to absorb the impact of disasters without loss of functions (termed **resistance**); maintain its most essential functions (called ***absorption and responsiveness***); and ‘bounce back’ to the pre-event state (termed **recovery**) or to a new state of function (termed **adaptation**).’  **Adaptive capacity** is an essential part of resilience and is defined as the ability of an organization to **alter** its strategy, operations, management systems, leadership structure and decision-support capacity to **withstand disasters**, generally by **adopting adaptive qualities and proactive responses**    **Adapted from MCEER’s framework to include four criteria; 4Rs:** ***Robustness*** (inherent strength to resist, withstand or absorb the shock), ***Redundancy*** (replace ability of resources), ***Resourcefulness*** (having plans and strategies) and ***Rapidity*** (achieve priorities promptly). The framework includes four criteria, two of which, **robustness and rapidity are seen as ‘ends’,** and two of which, resourcefulness and redundancy, are seen as ‘means’.  **Redundancy**: The extent to which elements of health facilities or health systems, that can be *substituted* for maintaining health functions  **Resourcefulness**: The ability to *identify problems, establish priorities*, and *mobilize resources* when disaster occurs | Healthcare resilience, especially hospital resilience, provides ‘lifeline’ services (i.e. continuity of service delivery) which ***further minimizes the impact*** of disasters on the community, and ***achieves higher community resilience.***  **Robustness**: Strength or the ability of health facilities or health systems to *withstand a given level of external shock*, and the extent to which the healthcare functions can be maintained  **Rapidity**: The *speed (in a timely manner)* of health facilities or systems with which the level of their full operational function can be achieved through the activities of *responsiveness, recovery and adaptation* |
| (18) | 3 x 4 matrix: 2 dimensions, 7 sub-domains, 22 factors:  To achieve resilience, it is essential to coordinate major related processes, resources, and technologies with the aim of managing expected and unexpected changes. It is also important to consider the main factors that can affect hospital resilience, preparedness, adaptive capacity, and rapid response to an unexpected incident.  22 influencing factors were included in a framework with 2 dimensions: (1) phases of the hospital resilience process (preparation, response, and recovery/growth) and (2) the key components of the hospital **(staff, infrastructure, management, and logistics).** | Utilizing Zhong et al.’s definition on hospital resilience: “the ability to resist, absorb, and respond to disasters’ consequences by providing additional services (e.g. on-site rescue, prehospital care, emergency treatment, critical care, decontamination and isolation), and then to recover or to adapt to a new situation.” | It minimizes the impact of disasters on vulnerable populations |
| (3) | Adapted from the **Hospital Safety Index**, this includes 2 areas: (physical and social), 3 domains (structural, non-structural, and functional/operational), 13-subdomains (Figure 4 of this article)  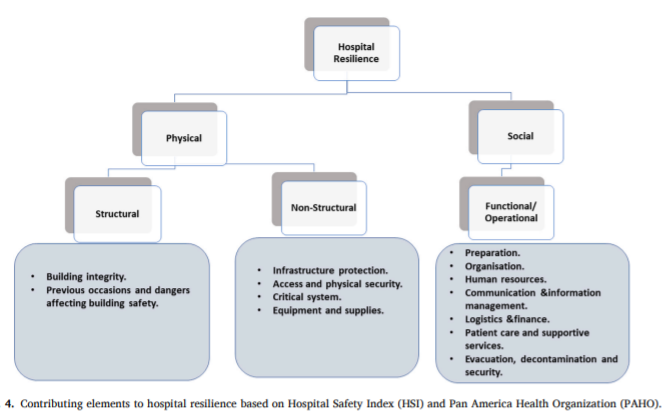  Requires considering the availability of disaster resources, disaster medical care, equipment capabilities, cooperation and training management, hospital safety, and disaster management mechanisms | Proposes a **4-step hospital-level disaster management plan/cycle (14 actions):**  1) Develop the plan,  2) Disseminate and communicate the plan,  3) Implement during the disaster, and  4) Update the plan (post-disaster)  for 3 functions: anticipate, respond to, and monitor disasters ***(See Figure 3 of this article)***  Utilizing the ‘plan-prepare-respond-recover’ (PPRR) model, and resilience engineering regarding four capacities: ‘Potential’ (to **anticipate** future threats and opportunities);  ‘Actual’ (to **respond** to events); ‘Critical’ (to **monitor** ongoing developments); and ‘Factual’ (to l**earn** from past failures and successes)  Focus on response: **Potential capacity** to anticipate and respond to disasters and **actual capacity** (performance) in responding.  ‘Improving resilience’ = improving the capacity of a hospital to respond, and the effectiveness of its response efforts during a disaster 🡺  **(3 types of resilience capacities: absorptive, adaptive, and restorative)**  These resilient systems or organizations have the capabilities of **being robust, flexible, and mannerly** in terms of: 1) responding to threats; 2) monitoring the current situation, including its performance; 3) predicting risks and opportunities; 4) notification of the outcome of events; and 5) **learning from experience** | High level of ‘business continuity’ – i.e. to be accessible at all hours, and to provide medical care, water,  food, power, and sometimes also information about the disaster itself, accommodation options, and about missing family |
| (19) | Hospital dimensions were referred to at the following frequency: **1) planning, management, and security, with most studies focusing on protection protocols; 2) human resources with most studies focusing on professionals’ well-being and mental health; 3) information and communication; and 4) finance**  No article specifically focused on the “governance” dimension (i.e., on hospitals’ leadership decisions); some mentioned elements concerning governance in studies that focused on the 4 other dimensions. | “The capacities of dimensions/components of a health system faced with shocks, challenges/stress or destabilizing chronic tensions (unexpected or expected, sudden or insidious, internal or external to the system), to absorb, adapt and/or transform in order to … (🡪) | … maintain and/or improve access (for all) to comprehensive, relevant and quality health care and services without pushing patients into poverty. |
| (21) | 3 categories **(human resources, management and communication, and the hygiene-security planning nexus)** and 10 conceptual dimensions of the framework: governance, intervention level, workforce, culture and social values, finance, planning and supported guidance, systems specificities, health sector management, information systems, context and security. | Same as above and using framework adapted from Ridde et al. 2021 (Figure 2 of this article) and WHO’s operational framework for building climate resilient health systems 2015 (Figure 2 of this article)  3 Hospital Resilience Processes: Absorption, Adaptation, Transformation  4 Hospital Resilience Outcomes: Collapse, Deteriorate, Recover, Improve  5 Impacts on Health Access: 5As Approachability, Acceptability, Availability, Affordability, Appropriateness. | |

## Annex 4: Capacities in systematic and scoping reviews on hospital and health systems resilience

|  | Absorb | Adapt | Transform | Learn | | |  |
| --- | --- | --- | --- | --- | --- | --- | --- |
| KK | Preparedness | Response | Recovery and Growth | | | |  |
| Zhong | Resistance: Capacity to absorb the impact of disasters without loss of functions | Absorption and Responsiveness: Capacity to maintain its most essential functions (e.g. prehospital care, emergency medical treatment, critical care, decontamination and isolation) | Recovery: Capacity to ‘bounce back’ to the pre-event state and/or  Adaptation: Capacity to a new state of function | | | |  |
| MohtadyAli | Absorptive Capacity: The system can withstand disruption | Adaptive Capacity: The system can use alternate reserves or processes while providing services (including monitoring performance, predicting risk, and reporting relevant information) | Restorative Capacity: Recovery from a disruptive event can be achieved rapidly and at sensible cost |  | | |  |
|  | ‘Potential’ (to anticipate future threats and opportunities);   ‘Critical’ (to monitor ongoing developments); | ‘Actual’ (to respond to events); |  | ‘Factual’ (to learn from past failures and successes) | | |  |
| WHO/EMRO Policy Paper | absorb the unforeseen shocks of an emergency, | adapt and respond to the emerging immediate and acute needs of the community, maintain its core functions, ensure the continuity of essential health services and deliver efficient, safe, high-quality, and person-centered care, | transform to recover, reduce vulnerability, and improve its readiness for future crises. |  | | |  |
| Iftaiel/Hollangel | 1.Anticipate, 2. Monitor | 3. Respond | | 4. Learn from | | |  |
| Biddle | Absorptive -- ‘capacity of a health system to continue to deliver the same level (quantity, quality and equity) of basic healthcare services and protection to populations despite the shock using the same level of resources and capacities’ | Adaptive -- ‘capacity of the health system actors to deliver the same level of healthcare services with fewer and/ or different resources, which requires making organizational adaptations’ | Transformative -- ‘the ability of health system actors to transform the functions and structure of the health system to respond to a changing environment’ |  | | |  |
| Foroughi et al | Absorptive strategies protect the system against shocks and the impact of hazards, which are usually small-scale shocks or events. These strategies return the system to its original state or reduce the severity and implications of the crisis on the system without making any particular change in structure, using available resources and capacities (skills, knowledge, tools, and data). Such strategies are generally used in the response phase. | | Transformative strategies or systemic resilience create long-term and significant changes in the system structure and functions in response to massive environmental changes or challenges. |  | |  |  |
|  |  | Adaptive strategies, named structural resilience, can lead to a limited number of gradual adjustments in the structure or process of the system. These strategies promote service delivery at the same level before the crisis and maintain core system activities using less or different resources Adaptive strategies will apply for more intensive challenges which absorptive strategies can’t deal with. |  |  | | |  |
| WHO/EURO | Ability to prepare for shocks— 1. Preparedness stage (before the shock onset): the system needs to get ready for shocks before they happen and identify optimal responses 2. Shock onset and alert: timely identification of the onset and type of the shock, which requires robust and comprehensive surveillance and early warning systems. | Manage (absorb, adapt, and transform) shocks (Shock impact and management) |  | Recovery and learning from shocks- when the shock has disappeared and there is a return to some kind of normality, there may also be useful learning from the shock experience and its management, not only for improving the current system but also in relation to better handling of any future similar shock scenario. | | |  |
|  | Manage (absorb, adapt, and transform) shocks (Shock impact and management) 1. Absorption: relates to incurring the system shock but protecting the health system from profound resource imbalance by making available additional resources, either from reserves or contingency planning 2. Adaptation: requires absorbing the additional demand or reduced supply, or both, by making the system more efficient (i.e. ‘doing more with less’ or by changing the allocation of resources). This may be a case of adapting delivery within the system 3. Transformation: the system may well need to change more fundamentally (transform) to cope with the impact of the shock. This may require a model radical rethinking of health system policy and the resourcing and delivery of care. This transformation process can sometimes compete with adaptation in relation to scarce governance capacity. | | | |  | | |

## Annex 5: Hospital resilience measurement tools across the literature

| **Article** | **Type of hazard** | **When?** | **Measurement or assessment strategies, tools, etc…** | **Donabedian** |
| --- | --- | --- | --- | --- |
| Zhong et al. (2013 and 2014) | All-hazard | Not specified | Adapted from MCEER; 4 Rs; 23 indicators across the 4 main domains (including: Emergency Medicine Response Capability, Disaster Management Mechanisms, Disaster Resources, and Hospital Infrastructural Safety)   - **Robustness** describes a hospital’s inherent strength to withstand the consequences of an event. - **Redundancy** is achieved through backup and surge capacity of staff, infrastructure, resources and equipment. - **Resourcefulness** is an adaptive flexibility for maintaining hospital essential services. - **Rapidity** reflects the speed of hospital responsiveness through fixing things up, bouncing back, functional recovery and adaptation. | Cap |
| Fallah-Aliabadi et al. (2020) | All-hazard  (disasters) | Not specified | ***Four measurement tools included each with their domains and indicators along with other indicators (see Table 2 in this article):*** HSI (structural, non-structural, functional); 2. Indicators for Assessing Hospital Disaster Preparedness in Japan (structural, non-structural, functional, human resources), 3. System Dynamics Approach to the Seismic Resilience Enhancement of Hospital (Endogenous, Exogenous, Excluded), 4: Zhong et al.’s Framework for Measuring the Hospital Disaster Resilience (Vulnerability and Safety, Disaster preparedness and resources, continuity of essential services, and recovery and adaptation)  Adapting HSI and Zhong et al. where the *proposed* ***constructive, infrastructural, and administrative resilience*** *domains and subdomains have the potential to substitute the 4Rs: resourcefulness, redundancy, robustness, and rapidity.* | ***Comp*** |
| Takim et al. (2016) | All hazards | Not specified;  For planning before building new facilities in risk-reduction stage | Developed hospital disaster resilience questionnaire: **Validated the 23 hospital resilience indicators proposed by Zhong et al. and the 129 hospital disaster preparedness proposed by HSI**  The disaster resilience assessment instrument comprises of 129 elements with three main constructs (i.e., 5 structural items -22 elements; 5 non-structural items - 43 elements; and 10 functional items - 64 elements). **48 out of 129 items were regarded as the utmost critical** by the content experts (including: structural - 2 items; non-structural - 7 items; and functional - 39 items; See table 6 in this article)  **This study analyzed 7 hospital assessment instruments, of which 6/7 were related to all-hazards:** 1) Health Facility **Seismic** Vulnerability Evaluation: A Handbook (WHO/EURO, 2006); 2) Field Manual for Capacity Assessment of Health Facilities in Responding to Emergencies (WHO/WPRO, 2006); 3) Hospital Safety Index: Guide for Evaluators (WHO/PAHO, 2008); 4) Hospitals Should be Safe from Disasters: Reduce Risk, Protect Health Facilities, Save Lives (WHO/WPRO, 2008); 5) Safe Hospitals in Emergencies and Disasters: Structural, Non-Structural and Functional Indicators (WHO/2010); 6) Hospital Safety from Disasters (Ardalan et al., 2014); 7) Hospital Disaster Preparedness Indicators (Bajow et al., 2014) | Comp |
| Goncalves et al. (2019) | All-hazards | Not applicable | A short-form version of the **Benchmark resilience tool for assessing the resilience of healthcare organizations**, explores two primary domains: **planning and adaptive capacity and 13 areas:**  1. We are mindful of how a crisis could affect us  2. We believe emergency plans must be practiced and tested to be effective  3. We are able to shift rapidly from business-as-usual to respond to crises  4. We build relationships with organizations we might have to work with in a crisis  5. Our priorities for recovery would provide direction for staff in a crisis  6. There is a sense of teamwork and camaraderie in our organization  7. Our organization maintains sufficient resources to absorb some unexpected change  8. People in our organization ‘‘own’’ a problem until it is resolved  9. Staff have the information and knowledge they need to respond to unexpected problems  10. Managers in our organization lead by example  11. Staff are rewarded for ‘‘thinking outside the box’’  12. Our organization can make tough decisions quickly  13. Managers actively listen for problems | Cap |
| Paterson et al. (2014) | Climate change | Planning and Preparedness | **A toolkit for assessing the resiliency of healthcare facilities in the context of climate change, including:**  **1. General climate change resiliency indicators for health care facilities** (e.g. cost-effectiveness of measures to address risks, opportunities to learn and increase awareness on climate change and sustainable practices, knowledge capacity, building adaptive capacity, strong leadership, and allocation of staff roles and responsibilities)  2.Emergency **Management** and Strengthening Health Care **Services** (in general, and for various types of emergencies such as: Extreme Weather, Food-borne and Water-borne Diseases and Outbreaks, Infectious Diseases, Air Pollution)  3. Climate Proofing and Greening **Operations**  The checklist has 82 questions within **four broad areas**: general information (n = 4), assessing climate-related **risks** (n = 19), **risk management** (n = 45) and building **capacity to adapt** to climate change (n = 14) | **Cap** |
| Mohtady Ali et al  (2021) | All-hazards | Post-disaster Evaluation | 3 main categories of post-disaster evaluation tools:  1. **Hospital functionality,** explored through 3 measures: 1.**1 Fault tree analysis** (can be a tool to estimate the loss of function of hospitals), 1.**2 Time-based** (the **quality** of provided care as measured through the waiting time. This index is correlated to the final loss of healthy people, caused by the healthcare facility performance during a disaster), and 1.**3 Crowdedness-based** (Emergency Department Over crowdedness which may decrease the **quality** of care provided; suggested tool: National Emergency Department Overcrowding Scale (NEDOCS) score).  2. **Hospital Safety** using the 151 indicators proposed by HSI across the following domains: structural, non-structural, functional (some studies separated out the human resource component).  3**. Other resilience measures including:** Measuring overall hospital resilience (capacities outlined by 4Rs in Zhong et al. 2013 and 2014), disaster **planning** assessment tools (hospital emergency and disaster readiness checklists), and measuring the **adaptive capacity** (specifically looking at business continuity management) | All |
| Jacques et al (2014) | Earthquake | Post-disaster evaluation | A fault-tree analysis method is adopted to assess the functionality of critical hospital services based on three main contributing factors: staff, structure, and stuff. **Fault-tree analysis was used in this study to estimate the loss of function of hospitals by service area. This loss of function can be used to estimate the hospital’s ability to bounce back from a disaster (or its resilience) and manage hospital surge while maintaining healthcare delivery to its community.**   - **Service loss and reduction of critical and lifesaving clinical and support services**:   - Y indicates a loss of service, where R indicates a reduction in service, and N indicates no loss of service   - **Top events are chosen as failure or reduction of critical service areas within a hospital, included: surgery, emergency department, intensive care unit, in-patient ward, obstetrics ward, laundry, kitchen, medical records storage, radiology, and administration**. - **Measuring bed capacity and referrals**: Initial capacity (beds), Residual capacity (beds), In-patients at the time of the EQ, Discharged patients in first 48 hours, Transferred patients in first week | All |
| Rahmani et al (2021) | Urban Resilience | Not specified  Ongoing/ Anytime (Planning) | Multiscalar and multi-dimensional approach: The Urban Resilience Index (URI) was assessed using a customized version of the City Resilience Profiling Tool (CRPT) established by the United Nations Office for Disaster Risk Reduction (UNISDR). **This tool has 5 components/attributes, 12 criteria, and 36 indicators.** The components or attributes and their criteria include: 1) Physical Attributes (Site plan, Hard infrastructure, Built-up environment), 2) Functional Attributes (land-use zoning, socio-economic, transportation), 3) Spatial attributes (accessibility and development plans), 4) Organizational attributes (institutional structure, hospital level structure, hospital functional scale), and 5) Functional evolution (role changing). Each of these has specific indicators | Comp |
| Moitinho de Almeida (2021) | Earthquake | Throughout (4 months pre-event-6 weeks after) | 1. Using routine hospital data (six weeks before to four months after the earthquake, and defined four periods: pre-earthquake (pre-EQ), acute (EQ1), post-acute (EQ2), and post-earthquake (postEQ)) **🡪 Hospital Admissions:** Time to event analysis (time to discharge or length of stay), number of daily admissions, diagnostic categories, and Hazard Ratios 2. Qualitative interview with staff about hospital resilience (adapting Zhong’s 4 R model) as it relates to health services provision and material challenges; quality of care and individuals/health workforce resilience | Out |
| Shang et al (2020) | Earthquake | Preparedness [Recovery plans] | 7 critical hospital sub-systems, 38 components   - The seismic resilience of an engineering system is often assessed using three indices, i.e., the recovery time, the economic loss, and the casualties (Cimellaro, Reinhorn, and Bruneau 2010a, 2010b). In this study, **the economic loss caused by the related components is used to quantify the corresponding functionality.**   A three-tier system model of a hospital system was developed and the importance factors of the different functional units, subsystems, and components were determined using a questionnaire provided to a multi-disciplinary expert panel. ). **The desirable recovery time of the hospital system was determined based on the questionnaire results and was used to evaluate the hospital resilience.** The resilience curve of the hospital system is quantified based on the **economic loss** weighted by the importance factors and the **recovery time** is determined based on the repair model. | All |
| Hassan and Mahmoud (2019) | Earthquake | Planning and preparedness | Hospital Functionality and Recovery measured **qualitatively** (patient’s satisfaction of the quality of service using **patient waiting time),** or **quantitatively** through **fault-tree analysis** estimated without considering interdependency on other lifelines. Adapted from Jacques et al, 3 hospital functionality domains (availability of staff, space and supplies) and **30 factors**; the **functionality recovery rate for different lifelines depends on the optimized repair resources**. | All |
| Abbasabadi Arab et al (2019) | All hazards | All stages | The Hospital Disaster Risk Management Evaluation (HDRME) consists of 8 constructs, including 7 enablers (management and leadership; risk assessment; planning; prevention and mitigation; preparedness; response, and recovery) and one result (key performance results). These constructs were further broken into 27 sub-constructs. Management and Leadership (3), Risk Assessment (2), Prevention and Mitigation (3), Preparedness (3), Response (6), Recovery (4), KPI Results (3). **The KPIs include organizational, staff, and society results** | N/A |
| Ridde et al (2021) | COVID-19 | Planning | 10 Dimensions of hospital resilience, simplified into three categories: 1) human resources, 2) management and communication, and the 3) hygiene-security planning nexus/  **3 Hospital Resilience Processes: Absorption, Adaptation, Transformation/ 4 Hospital Resilience Outcomes: Collapse, Deteriorate, Recover, Improve/ 5 Impacts on Health Access:** 5As Approachability, Acceptability, Availability, Affordability, Appropriateness. | All |
| Labarda et al (2017) | Typhoon | Retrospectively  Following the disaster | **Hospital resilience index,** derived from a self-reported checklist (Yes/No), adapted from Zhong et al. 2014’s: 8 Domains, and evaluate **102 indicators**. Each domain of hospital resilience measured key indicators that were scored either 0 or 1 given their absence or presence, respectively. A composite score for each domain was derived by summing all scores from the component indicators. Total scores were computed for both case hospitals from the sum of all domain scores. A hospital resiliency index was computed using the total score divided highest possible score. | **Com** |
| Romani et al (2021) | COVID-19 | During Response and Following | Modelling: 1**) risk of exposure and 2) % of ICU bed occupancy**. For risk: compartmental model SEIR, a classic infectious disease model that models the risks of exposure of people in 4 states: susceptible (S), exposed (E), infected (I), and resistant (R) and for bed capacity: current total number of ICU beds available in a catchment of 10,000 inhabitants | Out |
| Yin et al (2020) | COVID-19 | During Response and Following | **Qualitatively**: **Resilience Analysis Grid, notes four abilities of how a system responds, monitors, learns, and anticipates changing situations.** It was used as a frame of reference to guide observations, more descriptive, qualitative data such as how and why processes work out right, what improvisations look like, and what really happens on the ground as work-as-done instead of work-as-imagined. **Improvisations are cues to adaptive effectiveness and should serve to qualitatively measure resilience.** Improvisations can appear differently at various levels of resolution: organizational response, team performance, and individual improvisation. Improvisation is not simply allowing entities to act at will. Rather, it entails recognizing the tradeoffs, being aware of the risks, and where possible putting in place control measures. | **Cap** |
| Hansapinyo (2018) | Earthquakes | During | A questionnaire was distributed to the working people in the hospital. The information related to the important level of the lifeline system on the operation of the hospital was examined. There have been various kinds of rating scales developed **to measure the personal attitudes regarding the level of importance of the 7 lifeline systems**. Likert scale |  |
| Ramandi and Kashani (2018) | Earthquake | Response and Recovery (After disaster) | Two of the most important components of the research are hospital accessibility and capacity (affect patients’ waiting time). Other components are intensity of the hazard, quality of buildings and population of the region. These three components affect number of injured populations. We also consider number of ambulances, road condition and traffic condition as effective components of time on way to hospital. Finally, **quality of hospital performance is important in reducing waiting time and number of dead people.** **Resilience can be measured based on total dead number**. **Measuring**: a) Number of dead people before earthquake, b) Number of dead people after earthquake, c) Number of transferred patient before earthquake, d) Number of transferred patient after earthquake, e) Network waiting time before earthquake (min), f) Network waiting time after earthquake (min), g) Random treatment time (min). | Out |
| Norazam | Flooding | After | The investigation of hospital infrastructure resilience includes (i) **robustness** through building codes and structure, architecture, planning and zoning; (ii) **redundancy** through planning and operations; and (iii) **rapidity** through communication, movement, and risk assessment. Measured through: Hazard, Vulnerability and Capacity Assessment (HVCA) including: Planning and Layout, Site and Access, Building, Water supply systems, Electrical Supply Systems, Communication System, Gas Supply System | Cap |
| Pishnamazzadeh et al (2020) | N/A | N/A | Using system dynamic approach, Hospital Resilience level is calculated by tabulating **four Key Performance Indicators (KPI) of hospitals’ resilience considered:** **consisting of patient satisfaction, patient waiting time, staff burnout and staff satisfaction** against five exogenous factors: number of available beds, decision authority, number of nurses, home-work interface, & role ambiguity. **Hospital Admission** is a key process at hospital which links the four identified KPIs to each other. **Improve hospital performance** by modelling the performance from resilience engineering perspective.  Two types of costs related to resilience: (1) systemic impact of disruption which shows the differences of performance index before and after disasters, and (2) total recovery effort which indicates the **costs** of recovery action that would be done after disruptions to return the system state to pre-disruption one. | Inp/Out |
| Shahverdi et al (2020) | Multi  (pandemicearthquake, Flood, MCI) |  | Using a discrete event simulation framework and underlying open queuing network conceptualization involving patient flows through 9 critical units to measure hospital performance. This study explored impacts on critical resources, physical spaces and demand are modeled and the hospital system's resilience to these hazard events. The measure is Daily system-wide unmet demand, including the number **of patients left without being seen and expired patients** (together referred to as the unmet demand) and average **waiting times** are computed over 7 days. | Out |
